# Comprehensive Meta-analysis of Ontology Annotated 16S rRNA Profiles Identifies Beta Diversity Clusters of Environmental Bacterial Communities

**DOI:** 10.1371/journal.pcbi.1004468

**Published:** 2015-10-12

**Authors:** Andreas Henschel, Muhammad Zohaib Anwar, Vimitha Manohar

**Affiliations:** Department of Electrical Engineering and Computer Science/Institute Center Smart Infrastructure (iSmart), Masdar Institute, Abu Dhabi, UAE; University of Zurich and Swiss Institute of Bioinformatics, SWITZERLAND

## Abstract

Comprehensive mapping of environmental microbiomes in terms of their compositional features remains a great challenge in understanding the microbial biosphere of the Earth. It bears promise to identify the driving forces behind the observed community patterns and whether community assembly happens deterministically. Advances in Next Generation Sequencing allow large community profiling studies, exceeding sequencing data output of conventional methods in scale by orders of magnitude. However, appropriate collection systems are still in a nascent state. We here present a database of 20,427 diverse environmental 16S rRNA profiles from 2,426 independent studies, which forms the foundation of our meta-analysis. We conducted a sample size adaptive all-against-all beta diversity comparison while also respecting phylogenetic relationships of Operational Taxonomic Units(OTUs). After conventional hierarchical clustering we systematically test for enrichment of Environmental Ontology terms and their abstractions in all possible clusters. This post-hoc algorithm provides a novel formalism that quantifies to what extend compositional and semantic similarity of microbial community samples coincide. We automatically visualize significantly enriched subclusters on a comprehensive dendrogram of microbial communities. As a result we obtain the hitherto most differentiated and comprehensive view on global patterns of microbial community diversity. We observe strong clusterability of microbial communities in ecosystems such as human/mammal-associated, geothermal, fresh water, plant-associated, soils and rhizosphere microbiomes, whereas hypersaline and anthropogenic samples are less homogeneous. Moreover, saline samples appear less cohesive in terms of compositional properties than previously reported.

## Author Summary

We here set out to map the entirety of available environmental microbiomes in order to discover the underlying compositional characteristics. For us it is intriguing to see which environmental factors influence the assembly of microbiomes. We collected many diverse environmental samples and annotated them with a restricted, yet structured set of ecosystem terms. We then cluster all samples and automatically detect which ecosystems cluster together.

The resulting map provides an overview of relatedness between microbial communities from all types of ecosystems, their global patterns of diversity, i.e. their compositional similarities and differences. The comprehensive structure of relations provides insights to habitat adaptation and assembly rules.

Finally, utilizing the background database of samples we can now put new samples into an environmental context.

## Introduction

The often quoted tenet:“Everything is everywhere, but the environment selects” by Lourens Baas Becking has been subject to intense debate [1]. It gave rise to a series of hypotheses, how exactly the environment selects, i.e., which ecological rules are driving selection in which environment and whether they do so deterministically. The two competing theories for addressing this question are the ecological inference theory with a niche-based perspective [2, 3] and the neutralist random process theory [4]. Compact clusters of low beta diversity in microbial communities from the same environment indicate assembly determinism (i.e.), environmental factors predictably govern community composition. Conversely, if random processes and founder effects were the main drivers during community assembly, we would expect that this is reflected in high beta diversity and consequently low cluster homogeneity for samples from the same environment type. In order to elucidate these mechanisms as well as the environmental factors that drive bacterial community composition, it is necessary to develop a framework for comprehensive meta-analyses of microbial communities. To this end it is desirable to collect large, representative sets of samples from independent studies and diverse environments. In this light it is encouraging that the ever decreasing cost of DNA sequencing has led to a recent deluge of Metagenomics projects and Microbial Community profiling experiments in many, diverse ecosystems on the planet, e.g. the Human Microbiome Project and the Earth Microbiome Project [5, 6]. The primary data type for studying the community composition is the 16S rRNA gene, where hypervariable regions serve as phylogenetic markers [7]. Among the advantages of the 16S rRNA gene are the chronometric properties suitable for phylogeny construction and its widespread use to profile communities in all types of environments [8], i.e. to determine the relative abundance of the community members. Thanks to multiplexing and high read counts on Next Generation Sequencing (NGS) platforms, it is possible to generate a large number of samples with a single run on various recent platforms [7]. The 16S rRNA genes for many different bacteria have been sequenced and deposited in primary (GenBank) and secondary (GreenGenes, SILVA, RDP) databases [9–11]. However, community composition information, revealing co-occurrence of organisms, is lacking from these databases. On the other hand, microbial community collections such as those stored in the Sequence Read Archive [12] mainly focus on raw data deposition. MG-RAST [13] and CAMERA [14] (now defunct) are predominantly a repository for full shotgun Metagenomics. They do not maintain unified standards for Operational Taxonomic Unit (OTU) calling, i.e., the grouping of sequences into taxonomic levels of minimal sequence identity (commonly 97%). QIIME-DB (microbio.me/qiime) and the associated Global Environmental Sample Database (www.earthmicrobiome.org) are current efforts to overcome the above shortcomings but data deposition and retrieval methods are currently in a nascent stage.

Moreover, it is clear that the current community collections require formalisms to integrate metadata. Standards for data deposition (like “Minimum information about a marker gene sequence”, MIMARKS) have been introduced to address this problem [15], with ontology based knowledge management systems being an integral part of this. The introduction of an environmental ontology for ecosystems and -subsystems enables the semantic grouping and comparison of environments in an entirely new way: for example corals, dugong feces, ocean water, brine pools can all be associated to marine ecosystems; a relationship not automatically recognizable from pure text annotation. As a result, we can compare environments on various levels of abstraction and determine how widespread ecosystem-specific OTU compositions are. Nevertheless, quality control of these submissions remains difficult, as submitters might not be aware of the entire ontology structure and thus make non-optimal choices.

Clusterability of microbial communities has been investigated in the Human Microbiome Project, and various techniques and results, e.g. enterotypes have been presented [16] and debated [17, 18]. The effects of clustering methodology, distance metrics and taxonomic level of OTU picking are of great importance for the process of detecting clear-cut clusters of microbial communities. Importantly, traditional clustering algorithms for microbial communities are “uninformed” with respect to meta-data: the decision of partitioning is solely based on clustering structure and coefficients derived from beta diversity distances, disregarding useful semantic clues that ontologies can provide. Our philosophy is to postpone the decision to find meaningful clusters *after* a traditional hierarchical clustering structure is produced and ontology information for samples is taken into account. This is achieved by correlating the clustering structure with environmental categories—a novel approach in the realm of microbial community analysis. It is conceptually similar to the CLustering Enrichment ANalysis (CLEAN) described in [19], which integrates clustering of genes and their membership in functional categories such as Gene Ontology in the context of gene expression. We systematically generate and analyze a series of hypotheses to identify the extent that environments deterministically govern microbial community assembly. Interesting cases are those where the clustering structures (reflecting OTU compositions) and the post-hoc added environmental annotations coincide. This indicates which environmental factors were responsible for community composition. On the other hand, discrepancies can be further reconciled by considering additional meta-data (pH value, temperature) reflecting environmental differences or stochastic processes.

The feasibility of microbial community profile comparisons in meta-analyses depends on a number of aspects, such as standardization steps and sequencing platforms. In [20], Caporaso *et. al* show that biological conclusions were highly reproducible across lanes, read directions and Illumina HiSeq and MiSeq platforms. Other meta-analyses have demonstrated that microbial community samples are comparable across studies and platforms [7, 21, 22].

Previously Lozupone and Knight studied global patterns of bacterial diversity on the basis of a data set that comprised 202 samples from 111 studies [23]. The authors postulated that salinity and human-association are major environmental factors that drive community composition. They manually assigned 15 distinct environmental categories to all samples. While our approach draws a great deal of inspiration from this work, we extend and automatize it in various ways so to minimize sampling bias and to cope with the current and expected volumes of input data. First, the mentioned previous studies were performed on a small set of independent studies with comparatively low sequence counts. In fact, modern NGS platforms allow sample sizes far beyond 50 sequences (as used in [23]). This is more suitable for diversity studies, as more low abundance OTUs are accounted for. Our data collection is orders of magnitude larger, thus adds rigor to clustering observations but also demands appropriate storage and retrieval systems. The acquisition does not rely on single sequence retrieval from GenBank but builds on emerging repositories dedicated for 16S rRNA profiles that also provide community information and metadata. Together they provide a more representative snapshot of the Earth Microbiome and hence allow a more differentiated review of the early hypotheses on community assembly. Second, instead of just 15 manually defined environmental categories, we here describe how to to annotate samples using a suitable ontology, namely the Environmental Ontology [24]. Finally, we propose an algorithm that conducts cluster analysis with > 10,000 samples including exhaustive testing for enrichment of environmental attributes, borrowing techniques from Information Retrieval (for a thorough introduction, see [25]).

## Methods

### Overview

**Fig 1:**
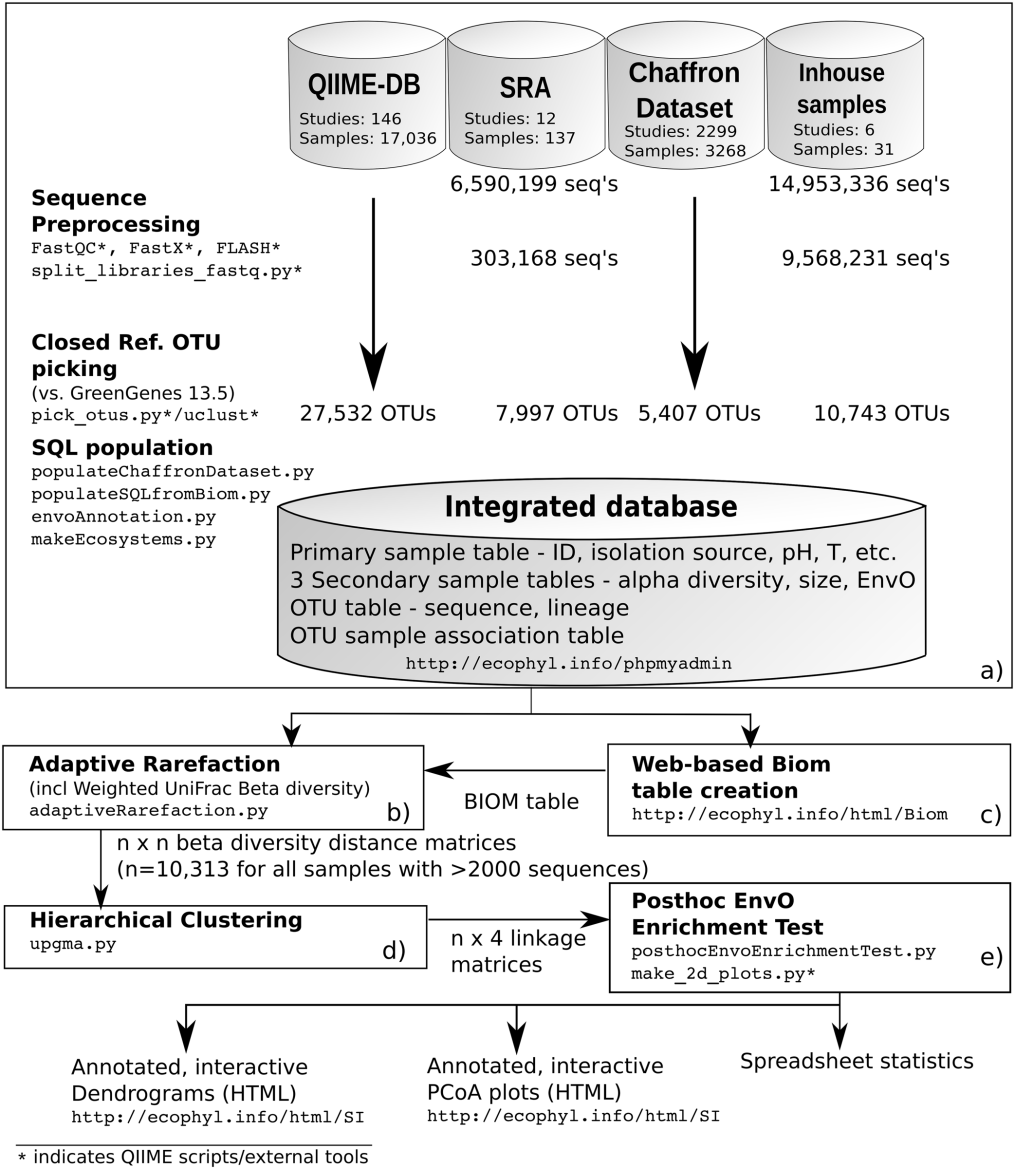
Overview of Microbial Community meta-analysis. The diagram lists all major components of our framework and their relation to each other. The results of each step are shown next to each component. Tools/scripts are shown in type writer font. a) Data acquisition and database creation: we collect data samples from four different sources and unify there representations such that they can be integrated in a single relational database. b) The given web page provides a user-friendly way to generate highly customized BIOM tables to facilitate user-specific meta-analyses. c) Adaptive rarefaction, taking input either from the database or from a BIOM table, produces all-against-all beta diversity distance matrices for the provided samples. d) Conventional hierarchical clustering. e) Posthoc enrichment test for EnvO annotations. The final output are a spreadsheet, documenting enriched clusters (precision, recall, F-measure, cluster coefficients, EnvO-terms, etc.), and interactive, annotated clustering visualizations.

The overview of all steps involved in our data acquisition and analysis is provided in Fig 1 displaying three major components: (i) an integrated, comprehensive database of OTU-clustered 16S rRNA profiles annotated with ontological metadata descriptors employing EnvO, the Environment Ontology [24], (ii) a module to compare microbial communities utilizing a phylogeny based distance measure ([26]) and Hierarchical Clustering (iii) an Information Retrieval based post-hoc cluster analysis to test for enrichment of EnvO terms in the clusters of the dendrogram.

### Data acquisition and Sequence Analysis

Large scale community comparisons must overcome innate sample differences such as uneven sampling size, different OTU calling methods and inconsistent or lacking environmental annotation. We composed a large meta-dataset from heterogeneous sources such as the QIIME-DB microbio.me/qiime/, Sequence Read Archive (SRA, [27]), a data collection provided by [28] (henceforth referred to as Chaffron dataset) and some locally sampled data. In total, we collected 20,472 distinct 16S rRNA from 2,461 different studies and stored them together with additional sample descriptions and meta data in a relational database (MySQL) for fast retrieval. Although the Chaffron data collection is composed of predominantly small samples (mainly non-NGS, lacking MIMARKS annotation), it proves to be valuable as samples are from 2297 independent studies, which contributes to the comprehensive nature of meta-analyses composed of global microbiomes and increases confidence beta diversity patterns. he sequence preprocessing includes: quality filtering, demultiplexing samples using QIIME’s split_libraries_fastq.py, and consistent closed-reference OTU calling against GreenGenes (version 13.5, 97% sequence similarity, using QIIME’s pick_closed_reference_otus.py). Closed reference OTU picking has a number of advantages (see also Discussion for caveats), namely it allows comparison of samples with different 16S rRNA regions, it comes with a high-quality phylogeny based on full-length sequences (thus facilitating phylogeny-based beta diversity calculation) and it is likely to filter chimera sequences. Also, for the sake of consistent data processing, note that UCLUST-ref [29] and GreenGenes were also used in the data sets acquired from QIIME-DB. According to [30], UCLUST-ref performs well in comparison to other reference and non-reference based methods. The projection of datasets on a limited set of reference OTUs incurs a loss in diversity. We therefore validate that the impact on beta diversity is within acceptable boundaries, i.e., beta diversity distances between open and closed reference OTU picking are correlated: for six environmental samples [31] where original sequences for the same 16S rRNA region are available, we compare beta diversity. FastTree [32] was used to construct the phylogeny for de novo OTU representatives, using QIIME’s make_phylogeny.py and pick_representatives.py with default parameters.

See Fig 1 for the different, data source specific steps and the overall workflow. To enable the integration with QIIME tools, we developed a web interface for custom BIOM table creation, a widely used format for ecological sample survey data [33]. The selection of samples is possible on the basis of EnvO annotation (including recursive sub-category) or presence/absence of lineages, metadata, and a combination of these criteria, thanks to a function for recursive EnvO traversal and the expressive power of SQL algebra.

### EnvO annotation and method validation

The aim of Ontology annotation is to use controlled vocabulary for different types of environments to hierarchically catecgorize samples so to be able to relate clusters of communities to environmental determinants. We used text-mining (weighted Jaccard Index for phrase similarity [34]) to automatically annotate samples lacking MIMARKS annotation (SRA, Chaffron’s dataset and our own) with EnvO-terms based on sample description texts like isolation-source: we regard both sample description and EnvO-terms as bags of stemmed words to accomodate word order permutations and inflections. The weighted Jaccard expressing the similarity of two phrases *A* and *B* is then given by:

$$J\left(A,B\right)=\frac{\sum_{w\in (A\cap B)}idf\left(w\right)}{\sum_{v\in (A\cup B)}idf\left(v\right)}$$ (1)

where *idf* is the inverse document frequency of a word with respect to the Brown corpus [35].

**Fig 2:**
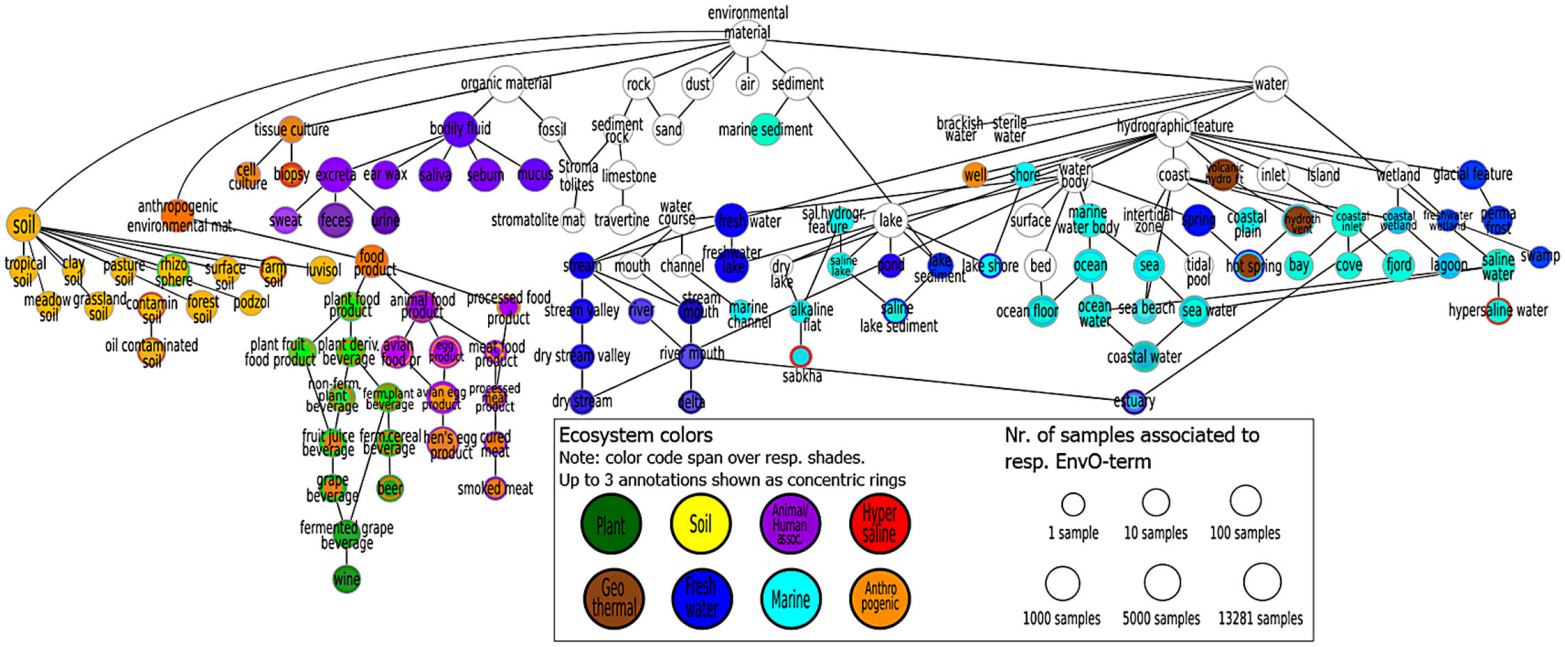
EnvO subgraph for environmental Material. Node size reflects number of samples assigned to the EnvO-term (logarithmic scale, see size legend, right). Node colors are shades of the overarching ecosystem color, see left legend. Multiple inheritence of EnvO-terms is reflected by several colors arranged in concentric rings.

EnvO is used by various projects [6, 13] to facilitate a principled approach towards environment classification by formalizing adequate naming conventions. It contains a rich, structured vocabulary (including synonyms), and it is arranged as a Directed Acyclic Graph, maintaining a general-to-specific order. We obtained the obo version of EnvO from obofoundries.org. We extend EnvO by creating new subclasses with terms that best describe environments of our database, including for example the human body site descriptions of the Human Microbiome Project. We also introduce missing semantic relationships, for example we describe “feces” (ENVO:00002003) as part of the gut (ID:0000002) to connect samples annotated with these respective terms that would otherwise appear unrelated. Multiple EnvO-terms can be associated to a sample, describing the biome, environmental feature and environmental material. We generated a subgraph for those EnvO-terms for which we found associations to microbial community samples. The graph coloring recursively assigns shades of the overarching ecosystem to its child nodes, while also reflecting multiple inheritence (Fig 2).

In order to programmatically make use of EnvO as a knowledge management system, we created a simple text parser that transforms obo-format into a richly annotated graph structure using networkx [36]. We also develop an API that lets us navigate along the graph structure. For validation of our annotation algorithm, we predict the best EnvO-term based on Eq 1 for 712 non-redundant sample descriptions and their annotations from all EnvO annotated QIIME-DB studies. We then calculate the minimal distance of the predicted and the manually annotated EnvO-terms (given in QIIME-DB as Environmental matter, Environmental feature and Environmental Biome). The distance is the shortest path in the undirected EnvO graph, such that exact matches have graph distance 0, EnvO-terms in direct subclass-superclass relation have distance 1, direct sibling nodes 2, cousins 4, etc. The results are shown in S5 Fig. It can be seen that from the 712 samples, most automatic annotations are in exact agreement (294) or in direct sub/superclass relation (117) with the manual annotation. By statistically summarizing all possible 7,122 graph distances in the EnvO graph, it turns out that the probabilities for two random nodes to be 0, 1, 2 and 3 steps apart are 0.06%, 0.20%, 1.18% and 3.42% respectively. Thus 73.3% of our annotations would have been predicted by chance with less than 3.5% probability. Note that manual EnvO annotations are a source of error as well and contribute to disagreement. We corrected for only very few blatant misannotations and consider therefore our accuracy estimates to be a conservative lower bound. Note that highlighting discrepancies can be used instructively during manual curation step in sample submission tools like QIIME-DB or MG-Rast.

### Definition of High level Ecosystems

Due to the multiple inheritence DAG (Multi-tree) structure of EnvO, it is possible to easily extend EnvO with further terms that capture high level concepts such as abstract ecosystems or environmental properties (saline, hypersaline environments, contamination and various subtypes of it, chemical enrichment such as nitrate, hydrocarbons). These overarching groupings also deal with EnvO’s attempt to include various, classification systems (including WWF, Udvardy and Baileys biomes). E.g., “Tundra”/“Tundra mire” or “Forest”/”Forest biome” are in entirely different branches of the ontology, despite the obvious semantic relation. We here propose high level ecosystems composed of distinct, yet related EnvO categories, see Table 1. Note that due to multiple inheritence and multiple annotations, a sample can fall into several ecosystems.

**Table 1:** Ecosystem definitions based on EnvO categories.

**Ecosystem**	**Subsumed EnvO-terms**
Plant	plantation, plant-associated habitat, plant food product, rhizosphere
Freshwater	freshwater wetland, glacial feature, reservoir, freshwater habitat, aquifer, fresh water, freshwater lake, freshwater biome
Soil	soil, mountain, mountain range, karst, terrestrial biome, plantation, mud, depression, pebble sediment, clay, terrestrial habitat, sandy sediment, landslide, beach, desert, subterrestrial habitat, sediment
Animal/Human	bodily fluid, animal food product, animal-associated habitat
Hypersaline	haline habitat, hypersaline
Marine	marsh, marine biome, marine sediment, saline water, coastal inlet, marine water body, saline hydrographic feature, coast, archipelago, marine channel, seashore, reef, undersea feature, black smoker, marine feature, marine snow, coastal wetland, saline water habitat
Geothermal	volcanic feature, geothermal power plant, volcanic hydrographic feature
Anthropogenic	anthropogenic feature, anthropogenic abiotic mesoscopic feature, anthropogenic environmental material, anthropogenic habitat, bioreactor, biofilter
Biofilm	biofilm, microbial mat material, biofilm material, microbial mat

### Adaptive rarefaction and Beta Diversity calculation

In order to compare samples it is highly recommendable to have comparable sample sizes. It is therefore common practice to apply rarefaction to samples that are to be compared, usually by randomly down-sampling. If we down-sample to the smallest sample size in the data set, this method unfortunately leads to a big loss of information when comparing samples of strongly differing sizes. E.g., representing communities of 10,000 different OTUs with only 10 or 50 OTUs neglects the majority of community members in the larger communities. On the other hand, if a larger target subsampling size is chosen for rarefaction, many small size communities have to be excluded.

We here device a method that for (almost) each pairwise sample comparison subsamples only to a size necessary for that individual pair, rather than subsampling all samples to the size of the smallest sample (as is commonly done with tools that perform rarefaction on BIOM tables, such as QIIME/UniFrac). By keeping the subsample size as large as possible, we increase the pairwise beta diversity distance precision. E.g., given 1001 samples *A*_1_ … *A*_1000_ of size 10,000 and *B* of size 1,000. We calculate beta diversity on subsamples of size 1,000 for *A*_*i*_-*B* and 10,000 for all pairs *A*_*i*_-*A*_*j*_. The latter, comprising 99.8% of all comparisons, would have been substantially less accurate, had we downsampled all samples to 1,000. We validate the claimed precision improvement by performing jack-knifing (multiple subsampling) on traditional one-size-fits-all and adaptive rarefaction: we repeat the random subsampling and beta diversity calculation ten times for 60 samples of five different size categories. For each repetition, distance matrices are calculated using Weighted UniFrac. Principal Coordinate Analysis (PCoA) and three-dimensional PCoA plots were produced with QIIME scripts principal_coordinates.py and make_3d_plots.py, respectively. Finally we calculate standard deviations for corresponding positions in the upper half of the distance matrix, convert it to a one-dimensional vector and apply the t-test for related samples (using ttest_rel from scipy.stats).

We calculate a complete distance matrix for all samples, effectively requiring n(n-1)/2 comparisons, with *n* = 10,313. In order to account for phylogenetic similarities of involved OTUs, we use Weighted UniFrac as beta diversity. We motivate this choice as follows: although the usability as a distance metric has been questioned in [37], the criticism was addressed in [22]. Moreover, it was shown to be an instance of the more general Earth mover’s (aka Kantorovich-Rubinstein) distance metric [38]. UniFrac has been applied in over 1500 research publications in a wide range of microbial community comparison tasks.

Note that our approach of retaining only closed-reference OTUs avoids the construction of phylogenetic trees, a very time consuming and error-prone task, by trimming a comprehensive, high-quality tree (provided by GreenGenes) to the relevant OTUs. It further enables phylogenetic beta diversity calculation of samples with different 16S rRNA regions. Subsequently we apply Unweighted Pair Group Method with Arithmetic Mean (UPGMA), a form of agglomerative Hierarchical Clustering, using SciPy.

### Alpha Diversity

We calculate alpha diversity using QIIME’s alpha_diversity.py for each ecosystem independently, considering both phylogenetic (Phylogenetic Distance) and non-phylogenetic (Chao1, observed species) methods. Every sample is downsampled to a range of suitable sizes between 60 and 60,000 counts. The results are stored in the provided MySQL database.

### Post-hoc Enrichment test of Environment Categories in Beta Diversity Dendrogram

After applying hierarchical clustering to all samples, we systematically analyze the resulting dendrogram structure and subcluster constituents. Hierarchical clustering yields a dendrogram encoded as a (*n* − 1) × 4 linkage matrix. For each possible cluster, we systematically test, whether it is enriched in any EnvO-category. We quantify this intuition by calculating precision, recall and F-measure: these tools, borrowed from information retrieval, express how well samples from a certain category cluster. A cluster containing predominantly members from one EnvO-category receives a high precision value. On the other hand, high recall is achieved if most category members are also subsumed under a cluster. The pseudo code is provided in Algorithm 1 (available at https://goo.gl/70LsQi). Moreover we determine the cluster coefficiencts describing the compactness of a cluster. High homogeneity (high intra-cluster similarity) and separation (low inter-cluster similarity) are indicators for a distinct, compact set of samples [39]. Homogeneity is determined by average distance between all members of the cluster, separation is the average distance to all members outside the cluster.

### Validation of Post-hoc Enrichment Test

Our method can be best compared to the CLustering Enrichment ANalysis (CLEAN) score [19], where environmental samples are the equivalent to genes (clustered by gene expression levels) and Environment Ontology annotation corresponds to membership of genes in functional categories such as those from Gene Ontology. We therefore compare the F-measure to Fisher’s exact test, which is central to the CLEAN score: we test to what extend the Fisher’s exact test and F-measure coincide wrt. significance for a given contingency table. We perform an overarching grid search for the two significance thresholds for both tests. For each significance threshold setting we consider a set of representative category and cluster sizes, calculate all possible contingency tables and measure when each test would call this significantly enriched. Note that we define the two groups of the contingency table to be all samples belonging to a cluster and all those that do not, respectively. We then count the percentage of cases of (dis-)agreement between the two tests.

### Identification of Salinity related samples

We imposed the property “saline” on high level EnvO-terms such as “marine feature” (ENVO:01000031), “marine water body” (ENVO:00001999) and “saline hydrographic feature” (ENVO:00000017). Consequently all subsumed EnvO-terms (incl. saline lake, ocean, lagoon etc.) inherit this property. The database lookup for EnvO-sample associations then facilitated fast and convenient identification of salinity related samples. Those samples are then marked in Suppl. S3 Fig.

**Algorithm 1** Bottom-up algorithm to determine dendrogram-clusters enriched in EnvO-terms. The dendrogram is the result from the hierarchical clustering (UPGMA) and encoded as linkage matrix.

 LinkageMatrix = UPGMA(beta diversity Distance Matrix)

**for all** rows *row* in LinkageMatrix **do**

  form new cluster **c** bottum-up from two subclusters as specified in *row*

*homogeneity* = average distance (*sample*_1_, *sample*_2_) ∀*sample*_1_, *sample*_2_ ∈ **c**

*separation* = average distance (*sample*_1_, *sample*_2_) ∀*sample*_1_ ∈ **c**, *sample*_2_ ∉ **c**

**if** *homogeneity*/*separation* < *threshold*_*density*_ **then**

   Document dense cluster (**c**, *homogeneity*, *separation*)

**end if**

**for all** EnvO-terms **e** present in **c** **do**

$recall=\frac{\text{samples with}\;\text{e}\;\text{in}\;\text{c}}{\text{samples with}\;\text{e}\;\text{in}\;\text{total}}$

$precision=\frac{\text{samples}\;\text{with}\;e\;\text{in}\;c}{\text{samples}\;\text{with}\;\text{in}\;c}$

$F_{1}=2\cdot \frac{precision\cdot recall}{precision+recall}$

**if** *F*_1_ > *threshold*_*enrichment*_ **and** *studies*(**c**) > 1 **and** Ontology-Depth(**e**) > 1 **then**

    Document enriched cluster (**c**, **e**, *homogeneity*, *separation*)

    Color linkages of **c** in dendrogram according to **e**

**end if**

**end for**

**end for**

## Results

We here describe a microbial community analysis framework extending conventional pipelines by including three novel components, which all help to enable all-encompassing meta analyses of 16S rRNA samples: (i) the creation of a 16S profile database from heterogeneous sources, (ii) adaptive rarefaction to compare large sets of samples of strongly differing sizes with minimal loss of information, and (iii) a post-hoc clustering algorithm that tests for enrichment of environmental categories (and their respective abstractions) after conventional hierarchical clustering of phylogeny based beta diversities. As a result we obtain the hitherto most differentiated and comprehensive view on global patterns of microbial community diversity with automatically detected enriched subclusters. It provides indicators for environmental factors that drive community assembly. Fig 1 summarizes all steps in our framework.

### Relational Database of annotated microbial community profiles

The creation of a comprehensive relational database of 16S rRNA community profiles constitutes an early result of our work. The database integrates various heterogeneous data sources. In total, we collected 20,472 samples comprising 6,331,600 sequence-sample associations from 2,462 independent studies. 10,313 of these samples are of suitable size for diversity studies (i.e. > 2000 sequences, according to [7]). Performing closed-reference OTU picking against a consistent reference (GreenGenes 13.5) yielded 40,164 OTUs, corresponding to 40.44% of GreenGenes’ 97% sequence identity clusters. Our pipeline makes use of OTUs being from a closed reference as it allows fast, yet phylogeny-sensitive beta diversity calculations without reconstruction of phylogenies.

To estimate the loss of diversity incurred by closed reference OTU picking, we list the number of dropped sequences for inhouse samples in Supplementary S2 Table. It shows that also for the case of environmental samples, a substantial amount of sequences is retained. We also measure the impact on beta diversity and compare to the corresponding results from de novo OTU picking. The beta diversity distances of the two methods are strongly correlated (Pearson correlation 0.82), thus justifying closed reference OTU picking as a proxy (see further contemplations in Discussion).

An overview of the data integration steps (including results) is shown in Fig 1(a). The central tables of the database are for sample description (ID, isolation source, associated publication, further meta-data), OTUs (GreenGenes identifier, RDP lineage, sequence) and sample-OTU association. Further, the database contains various sample annotations (EnvO, meta-data, alpha diversity, ecosystem coloring, sample size). The database scheme is provided in Supplementary Material, S2 Fig. All tables are appropriately indexed. The database is freely accessible through http://ecophyl.info/phpmyadmin/ (*User login is provided upon request from corresponding author*) or downloadable as an SQL dump. We argue that this form of storage is a viable concept to integrate the current and anticipated amounts of microbial community data such that fast and powerful queries are possible. To elaborate meta-study compositions, the relational algebra of SQL allows to combine conditions on sample annotations and properties as well as OTU annotations. For example, it is straightforward to retrieve all samples of a given alpha diversity from all subtypes of marine environments containing OTUs belonging to the Vibrio genus. For identifying environment subtypes, we employ EnvO’s General-to-Specific ordering of environments arranged along a Directed Acyclic Graph. EnvO-terms that were used to annotate samples are shown in a color coded Ontology subgraph in Fig 2. Finally, our framework includes a tool for BIOM table creation upon sample selection (see Fig 1(b)) for customized meta-analyses using adaptive rarefaction or further integration with tools like QIIME [40].

### Increased Comparability of 16S rRNA profiles from different Sequencing Platforms through adaptive rarefaction

The acquired profiles stem from different sequencing platforms and hence differ strongly in sampling effort/sequencing depth. In order to calculate alpha and beta diversity for a collection of heterogeneous samples, it is common practice to subsample all samples using rarefaction to a size smaller than the smallest sample to be included. This one-size-fits-all rarefaction seems unsuitable for a large set of samples with strongly varying depth as it incurs either strong downsampling on large samples (which might yield non-representative subsets) or exclusion of many smaller samples below a certain threshold. To address this problem, we provide an algorithm that for each pairwise beta diversity calculation rarefies samples only to a size necessary for the individual pair at hand, rather than subsampling all samples to a size below the smallest sample to be included. We are able to show that adaptive rarefaction produces more accurate beta diversity distances: multiple subsampling repetitions (jack-knifing) with static and adaptive rarefaction lead to significantly smaller distance variances for adaptive rarefaction (as shown by Student’s t-test, *p* = 3.3 × 10^−258^, S4(c) Fig. We also observed that for nearly all cases the distances from adaptive rarefaction were strictly contained by the range of distances from static rarefaction. We also visualize this process using three-dimensional PCoA plots, where larger uncertainty ellipsoids for traditional rarefaction indicate larger variance, see S4(a) and S4(b) Fig. The runtime of the Adaptive rarefaction algorithm is *O*(*n* ^2^∣*P*∣), where *n* is the number of samples to be compared and ∣*P*∣ is the size of the reference phylogeny (the GreenGenes phylogeny, in our case).

The adaptive rarefaction component is shown in Fig 1(c). The main result of adaptive rarefaction is a high accuracy distance matrix for 10,313 samples.

### Identification of Alpha diversity distribution in different ecosystems and -subsystems

**Fig 3:**
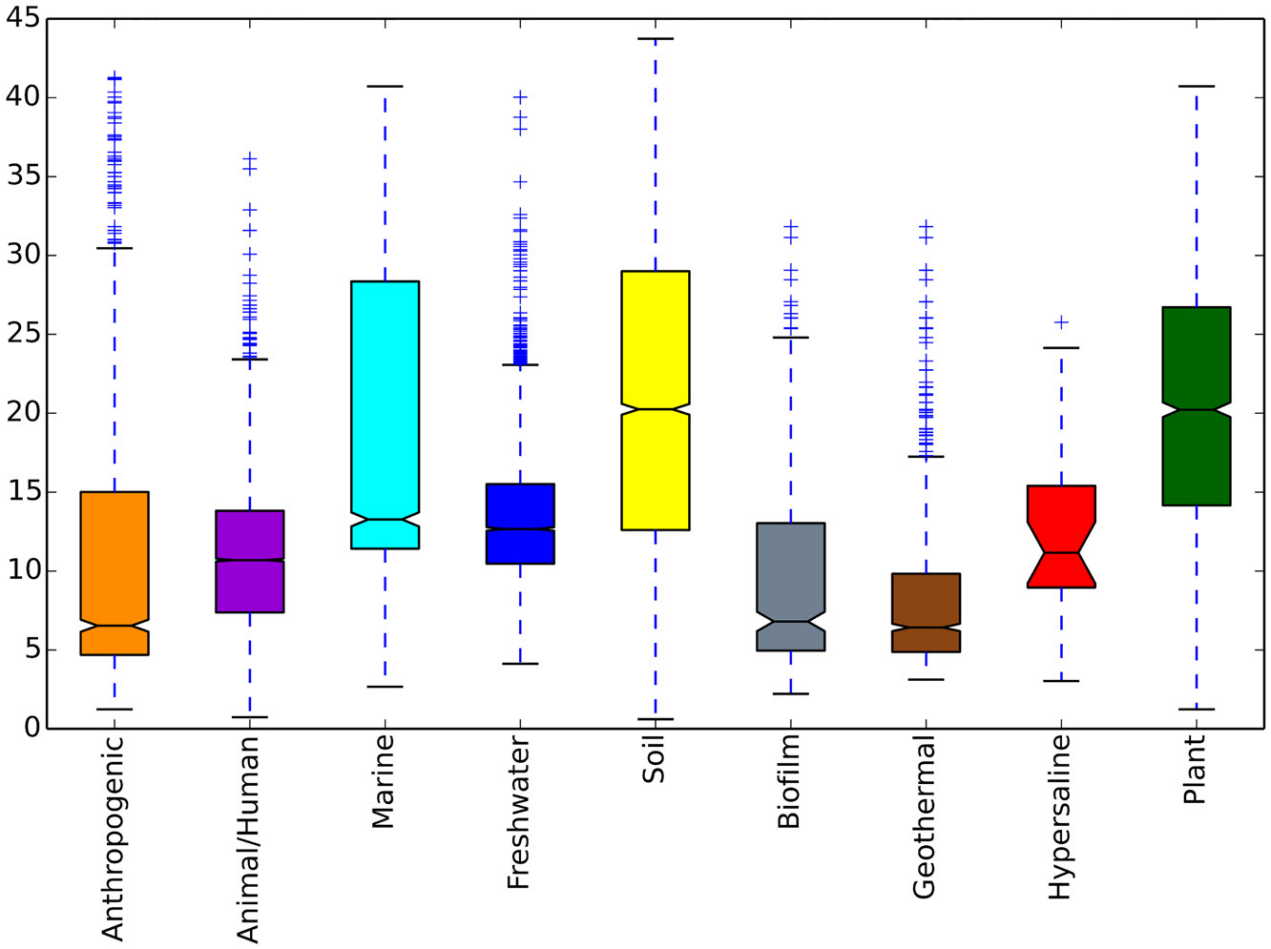
Alpha diversity box plots for different ecosystems. Based on our dataset, we observe that soil, marine and plant-associated environments in general host more diverse communities. Thanks to the applied sub-categorization, we can further break down ecosystems to inspect diversity in different soil types (shown in supplementary S1 Fig). We calculate Phylogenetic Distance Alpha diversity from samples rarefied to 1140 sequences.

Fig 3 shows that soil-, plant- and marine ecosystems are most diverse in terms of Alpha diversity. We can further break these findings down to subsumed environments. This reveals that among soil environments, farm soils are most diverse (Suppl. S1 Fig).

### Correlations and discrepancies of clustering structure with Environment Ontology enrichment indicate driving forces for community assembly

**Fig 4:**
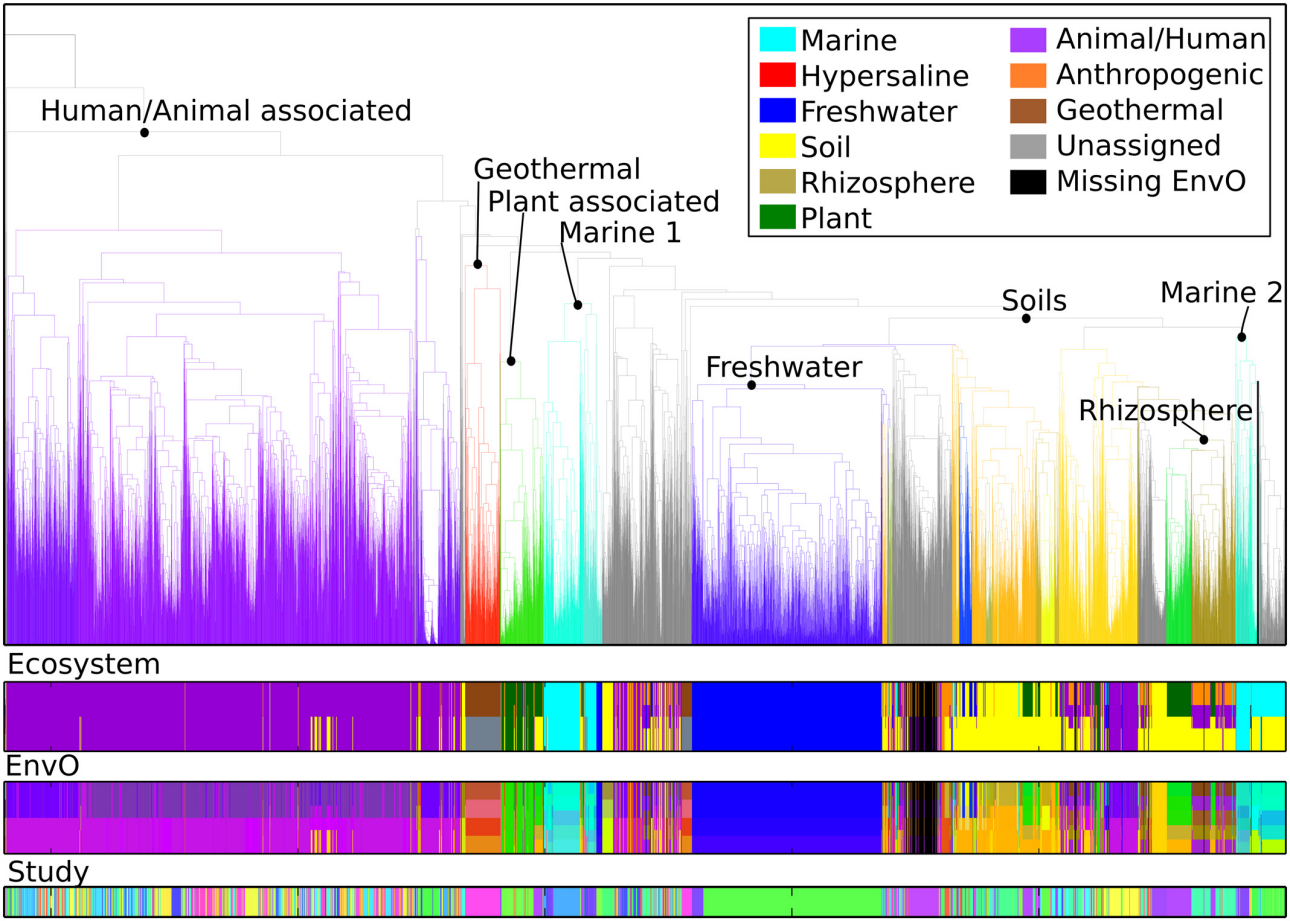
Comprehensive clustering of 10,313 samples with at least 2000 sequences. Clusters enriched in EnvO-terms are identified and color-coded automatically if *F*_1_-score > 0.5. Note that in the dendrogram, the entire clade is colored by the color of the enriched EnvO-term. The human/animal associated and soil clusters are supported by many independent studies, whereas freshwater and geothermal clusters are largely driven by findings of a single study. Study color, ecosystem colors and EnvO associations are visualized in the colorbars below the dendrogram. EnvO-annotation colors are shades of the associated ecosystem color (see legend).

**Fig 5:**
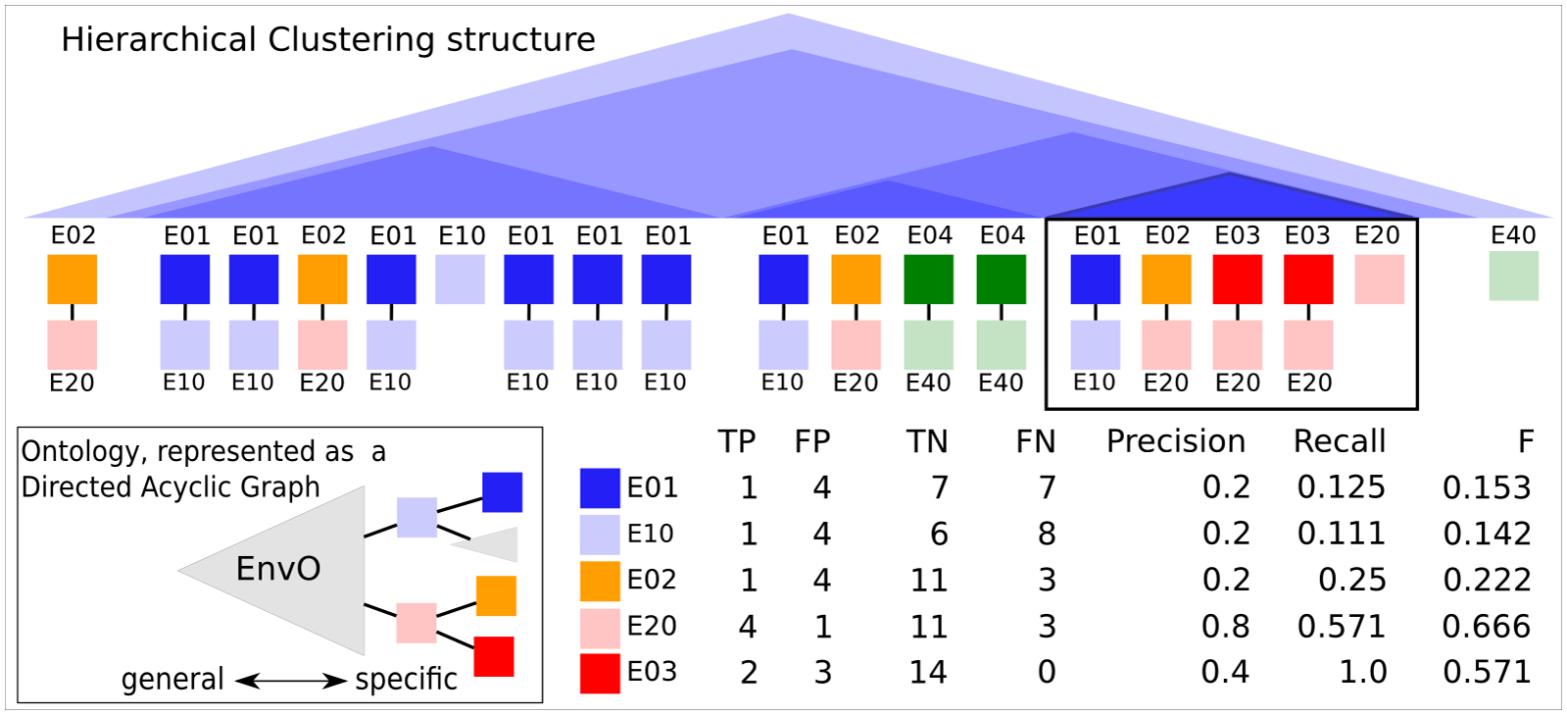
Illustration for Algorithm 1. Given a hierarchical clustering of samples that are annotated with Ontology terms (colored boxes, ancestry relations are shown with black lines), it detects enriched ontological categories on various levels of abstraction in each possible cluster: while analyzing the indicated cluster (black box, emphasized triangle), all present categories (and their ancestral categories) are characterized by their F-measure. E03 and especially E20 (parent of E02 and E03) are relatively specific for this cluster, as evidenced by a relatively high F-measure, whereas E01, E10 and E02 are mostly present outside the cluster, reflected by a small number of True Positives. Abbreviations: TP = True Positives, FP = False Positives, TN = True Negatives, FN = False Negatives.

**Fig 6:**
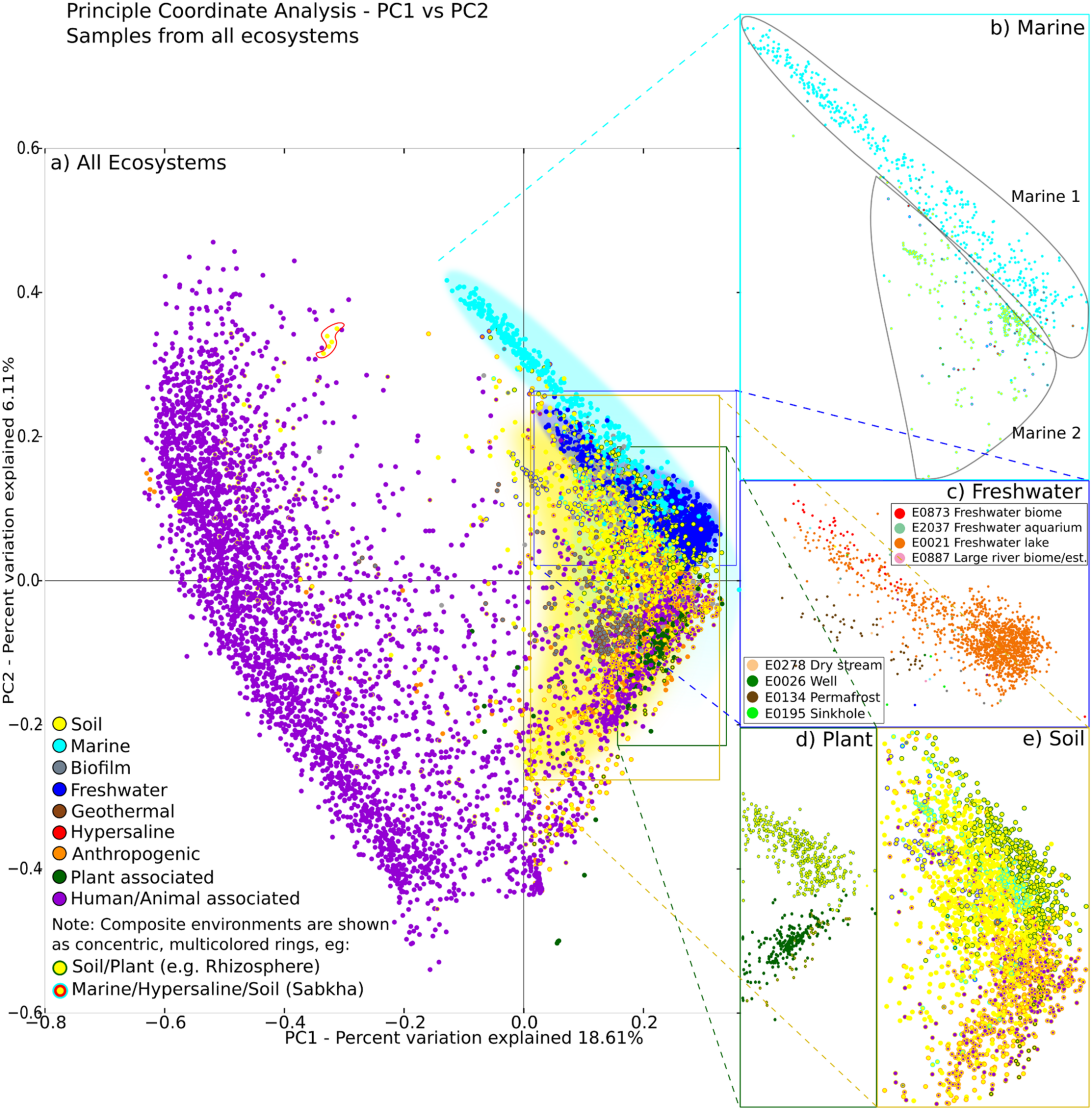
PCoA plot (principal components 1 and 2) for the same samples as in Fig 4. The scatter plot shows relatively cohesive and distinct ecosystems. While large studies often constitute the bulk of ecosystem clusters, detailed inspection shows support from further, smaller studies. Data points for certain ecosystems have been separated in the subgraphs b) to e). a) PCoA scatter plot including all samples from all environments. The first component largely separates human and and environmental samples, while the second component helps to identify clusters for soil, marine, freshwater and plant-associated samples. Misannotations of insect-associated samples (wrongly annotated as Soil) are shown in the red shape. b) The two main marine clusters, “Marine 1” and “Marine 2” (corresponding to the clusters in Fig 4 with the same name) are identifiable through the composite Ecosystem coloring: Marine sediments, shown in cyan/yellow mostly form “Marine 2” due to their dual membership in soil and marine environments; in contrast “Marine 1” samples are solely colored cyan. Hypersaline samples (red) appear widespread and non-cohesive. c) Fresh water samples, colored by Envo-ID. Several environments (freshwater biome, aquarium, freshwater lake) appear strongly related, while samples from permafrost and sinkholes are outliers. d) Plant samples split according to the two main contributing studies QiimeDB 1792 and 2019 respectively. Each cluster receives further support from small and medium sized studies. e) Soil samples. Composite environments form sub-clusters.

In order to test, whether a cluster is significantly enriched in environmental categories, we determine to what extend EnvO annotations —at all abstraction levels— are predominant in any part of the dendrogram, see Fig 4. We developed an algorithm that performs this task as a post-hoc clustering analysis, Fig 1(e), i.e, after Hierarchical Clustering Fig 1(d). The pseudo code is given in Algorithm 1 and a more detailed explanation is in Fig 5. It constitutes a rigorous formalization and implementation of the manual process outlined in [23], performed on tens of thousands of microbial community samples. It automatically identifies clusters enriched in environmental categories based on precision, recall, *F*_1_-score, number of studies and cluster coefficients homogeneity and separation (see Methods). The algorithm output of enriched clusters together with their respective EnvO-terms (*F*_1_-score > 0.5) is provided in Table 2. Ocean floor, bed (the portion of the ground surface which lies below water), grassland soil, small lake bioms, gut and animal associated habitat all are enriched in identified clusters and therefore seem to bear significant compositions that are driven by their respective environmental conditions. The color coding of these findings into the Hierarchical Clustering dendrogram shows that environmental samples cluster non-randomly (Fig 4). Although few inconsistencies persist, major ecosystems are recognizable, as soil samples, freshwater, rhizosphere, geothermal and the majority of human/animal-associated all fall together in respective clades of the UPGMA dendrogram. The clusterability of these environments is corroborated by visually inspecting Principal Coordinate Analysis plots, see Fig 6. There, the first Principal Component largely separates human and and environmental samples, while the second component helps to identify clusters for soil, marine, freshwater and plant-associated samples.

**Table 2:** Clusters from enriched in Environmental Ontology terms (as determined by).

EnvO ID	count	cluster	Total	Studies	Generality/EnvO-term	precision	recall	*F*_1_	homogeneity	separation
ENVO:00000501	200	208	206	3	4 bed	0.9615	0.9709	0.9662	0.2203	0.6828
ENVO:00000426	200	216	206	7	3 ocean floor	0.9259	0.9709	0.9479	0.2332	0.6882
ENVO:00000892	1532	1624	1615	16	3 Small lake biome	0.9434	0.9486	0.9460	0.3331	0.7169
ENVO:00002113	263	286	290	10	2 marine sediment	0.9196	0.9069	0.9132	0.3118	0.7045
ENVO:00000039	215	217	258	3	5 fjord	0.9908	0.8333	0.9053	0.2554	0.7411
ENVO:00002008	120	123	149	4	1 dust	0.9756	0.8054	0.8824	0.1371	0.6403
ENVO:00003003	15	19	15	3	3 Humid Tropical Domain	0.7895	1.0000	0.8824	0.1868	0.7585
ENVO:00000094	282	285	366	3	3 volcanic feature	0.9895	0.7705	0.8664	0.3768	0.7987
ENVO:00006776	3258	3300	4356	69	3 animal-associated habitat	0.9873	0.7479	0.8511	0.5849	0.8512
ENVO:02000022	2210	2731	2471	61	3 excreta	0.8092	0.8944	0.8497	0.5428	0.8429
ENVO:02000004	210	246	250	6	3 nesting material	0.8537	0.8400	0.8468	0.2694	0.6666
ENVO:00005778	26	39	26	3	2 tropical soil	0.6667	1.0000	0.8000	0.1131	0.7114
ENVO:00002024	10	13	13	3	3 haline habitat	0.7692	0.7692	0.7692	0.5724	0.8394
ENVO:00002151	359	477	479	20	3 ocean water	0.7526	0.7495	0.7510	0.4547	0.8031
ENVO:00002261	145	204	185	12	2 forest soil	0.7108	0.7838	0.7455	0.2359	0.6786
ENVO:00001998	787	833	1308	31	1 soil	0.9448	0.6017	0.7352	0.3770	0.7160
ENVO:00002150	88	96	145	4	4 coastal water	0.9167	0.6069	0.7303	0.3391	0.7597
ENVO:00002036	5769	10367	5779	133	1 habitat	0.5565	0.9983	0.7146	0.6864	0.8342
ENVO:00000000	3650	6647	3726	100	1 geographic feature	0.5491	0.9796	0.7037	0.5595	0.8032
ENVO:00005750	28	51	29	3	2 grassland soil	0.5490	0.9655	0.7000	0.1430	0.6402
ENVO:00002116	23	42	28	4	2 contaminated soil	0.5476	0.8214	0.6571	0.2843	0.6550
ENVO:00003982	16	22	29	4	4 travertine	0.7273	0.5517	0.6275	0.2647	0.6910
ENVO:02000036	286	509	430	21	3 saliva	0.5619	0.6651	0.6092	0.4368	0.7578
ENVO:00002016	17	24	32	6	2 sedimentary rock	0.7083	0.5312	0.6071	0.2744	0.6944
ENVO:00000134	49	96	66	3	4 permafrost	0.5104	0.7424	0.6049	0.2912	0.6553
ENVO:00000477	13	25	22	5	4 mount	0.5200	0.5909	0.5532	0.1426	0.6911
ENVO:00002875	13	19	28	3	3 oil contaminated soil	0.6842	0.4643	0.5532	0.1866	0.6417
ENVO:02000040	133	135	346	4	3 mucus	0.9852	0.3844	0.5530	0.0749	0.7994
ENVO:00000106	64	105	132	5	3 grassland	0.6095	0.4848	0.5401	0.1919	0.6726
ENVO:00000878	275	559	460	14	3 Mediterranean forests, woodlands,	0.4919	0.5978	0.5397	0.3036	0.6730
ENVO:00005801	241	462	444	12	2 rhizosphere	0.5216	0.5428	0.5320	0.2796	0.6684
ENVO:00009001	283	348	719	11	3 plant-associated habitat	0.8132	0.3936	0.5305	0.2796	0.7862
ENVO:00000875	6	15	8	4	3 Temperate coniferous forest	0.4000	0.7500	0.5217	0.1314	0.7054
ENVO:00000446	994	1428	2528	53	1 terrestrial biome	0.6961	0.3932	0.5025	0.4193	0.7395
ENVO:00000877	83	188	143	12	3 Temperate grasslands, savannas	0.4415	0.5804	0.5015	0.2774	0.6715

We regard clusters with *F*_1_-score > 0.5 as enriched in an EnvO-term and list them ordered by descending *F*_1_-score. We further report the count of samples associated to the dominating Envo-Term in a cluster (count), the cluster size (cluster), the total count of samples associated to the dominating Envo-term (Total), the number of independent studies (Studies), generality of an EnvO-term (Generality), measured as the hierarchical level in the Directed Acyclic Graph of EnvO, precision, recall, *F*_1_-score, homogeneity and separation. The list is filtered by EnvO-terms that appear at least in three independent studies.

Marine samples fall into two separate clusters, as shown by both UPGMA and PCoA. Cluster “Marine 1” is mainly composed of samples from QIIME-DB studies 1222 (Bergen Ocean Acidification Cosms), 1235 (Fjord mesocosms) and 1240 (Western English Channel time series). Interestingly, given the overall picture of beta diversity distribution, these marine samples cluster well despite their geographically different sampling locations. Further, small scale studies also fall into this cluster, see ecophyl.info/html/SI/PCOA/PCoA_Marine. Cluster “Marine 2” is composed of marine sediments/contaminated marine environments (QIIME-DB studies 1046, 1039 and 1198). It is remarkable that “Marine 2” appears closer to soils, again confirmed by both UPGMA and PCoA.

Plant samples also fall into two main clusters. Samples from QIIME-DB study 1792 (maize rhizosphere) appear close to marine samples in the PCoA plot (principal components 1 and 2), however, UPGMA separates them more clearly from marine samples. QIIME-DB studies 2019, 1690 and 1689 constitute the second plant cluster.

The human/animal-associated cluster is clearly separate from environmental samples: it has a separation score of 0.8512, the highest of all clusters. On the other hand it is not very homogeneous, as evidenced by large branches within the cluster and a homogeneity of 0.5849, i.e., it is among the least homogeneous of the detected clusters. Moreover, a smaller cluster of human/animal associated samples groups better with soil samples, possibly due to compositional similarities to soil samples with fecal contamination.

We also scrutinized the compactness of ecosystems by investigating cluster coefficients of all samples related to an EnvO-term, regardless of the clustering structure. We observe that hypersaline samples show the least homogeneity as compared to all other ecosystems, see Table 3. suggesting that these extremophiles get recruited predominantly through non-deterministic processes or unaccounted environmental factors.

**Table 3:** Cluster coefficients for homogeneity (cluster compactness) and separation for selected ecosystems and -subsystems (including all samples).

**EnvO**	**Ecosystem**	**Level**	**Homogeneity**	**Separation**	**Studies**	**Samples**
plant-associated habitat (ENVO:00009001)	Plant	3	0.4634	0.6816	4	744
organism-associated habitat (ENVO:00002032)	Plant	2	0.7001	0.7506	76	7582
habitat (ENVO:00002036)	Plant	1	0.7097	0.7198	117	9067
environmental feature (ENVO:00002297)	Plant	0	0.6872	0.7009	149	13281
plant food product (ENVO:00002216)	Plant	3	0.4558	0.7167	2	40
food product (ENVO:00002002)	Plant	2	0.4268	0.6508	5	565
anthropogenic environmental material (ENVO:0010001)	Plant	1	0.4268	0.6508	5	565
environmental material (ENVO:00010483)	Plant	0	0.6869	0.6955	145	12664
anthropogenic (ID:0000068)	Plant	1	0.5049	0.6648	27	1158
property (ID:0000043)	Plant	0	0.5651	0.6873	38	1863
soil (ENVO:00001998)	Plant	1	0.4726	0.6610	30	1438
wetland (ENVO:00000043)	Freshwater	3	0.4280	0.6345	2	13
hydrographic feature (ENVO:00000012)	Freshwater	2	0.5288	0.7070	32	3467
water (ENVO:00002006)	Freshwater	1	0.5321	0.7084	36	3551
geographic feature (ENVO:00000000)	Freshwater	1	0.5456	0.7133	54	3927
freshwater habitat (ENVO:00002037)	Freshwater	3	0.3676	0.6734	2	34
aquatic habitat (ENVO:00000144)	Freshwater	2	0.4990	0.6760	3	43
fresh water (ENVO:00002011)	Freshwater	2	0.3440	0.6730	7	1656
freshwater lake (ENVO:00000021)	Freshwater	3	0.3395	0.6727	4	1625
lake (ENVO:00000020)	Freshwater	4	0.3481	0.6733	9	1658
water body (ENVO:00000063)	Freshwater	3	0.5244	0.7040	25	3256
freshwater biome (ENVO:00000873)	Freshwater	2	0.3629	0.6755	10	1735
aquatic biome (ENVO:00002030)	Freshwater	1	0.5172	0.7053	22	2881
biome (ENVO:00000428)	Freshwater	0	0.5613	0.7511	60	5743
physiographic feature (ENVO:00000191)	Soil	2	0.5604	0.7133	11	608
terrestrial biome (ENVO:00000446)	Soil	1	0.5529	0.6836	41	2862
depression (ENVO:00000309)	Soil	3	0.4755	0.6487	2	22
terrestrial habitat (ENVO:00002009)	Soil	2	0.4917	0.6651	32	1602
desert (ENVO:00000097)	Soil	3	0.3239	0.7154	2	102
extreme habitat (ENVO:00002020)	Soil	2	0.5223	0.7194	6	164
subterrestrial habitat (ENVO:00000572)	Soil	2	0.4795	0.6914	5	192
sediment (ENVO:00002007)	Soil	1	0.3874	0.6512	7	339
bodily fluid (ENVO:02000019)	Animal/Human	2	0.6760	0.7707	67	6247
organic material (ENVO:01000155)	Animal/Human	1	0.6762	0.7798	79	6978
animal food product (ENVO:0010000)	Animal/Human	3	0.4049	0.6449	3	525
animal-associated habitat (ENVO:00006776)	Animal/Human	3	0.6714	0.7819	70	6813
hypersaline (ID:0000111)	Hypersaline	2	0.6918	0.8661	3	14
“saline feature” (ID:0000110)	Hypersaline	1	0.4813	0.7086	11	705
marine biome (ENVO:00000447)	Marine	2	0.4457	0.6768	7	370
marine sediment (ENVO:00002113)	Marine	2	0.3387	0.6483	5	292
saline water (ENVO:00002010)	Marine	2	0.4660	0.7276	9	502
coastal inlet (ENVO:00000137)	Marine	4	0.3916	0.7099	3	299
coast (ENVO:00000303)	Marine	3	0.4591	0.7005	5	404
inlet (ENVO:00000475)	Marine	3	0.3916	0.7099	3	299
marine water body (ENVO:00001999)	Marine	2	0.4690	0.7054	9	690
saline hydrographic feature (ENVO:00000017)	Marine	3	0.4989	0.7799	2	8
watercourse (ENVO:00000029)	Marine	4	0.4386	0.6720	3	13
marine feature (ENVO:01000031)	Marine	1	0.4691	0.7053	10	691
volcanic feature (ENVO:00000094)	Geothermal	3	0.4557	0.7276	2	366
anthropogenic feature (ENVO:00000002)	Anthropogenic	2	0.5475	0.6653	20	525
mesoscopic physical object (ENVO:00002004)	Anthropogenic	1	0.4522	0.6467	5	800
anthropogenic habitat (ENVO:00002031)	Anthropogenic	2	0.5848	0.7171	11	202
biofilm (ENVO:00002034)	Biofilm	3	0.5441	0.7376	3	38
microbial feature (ENVO:01000007)	Biofilm	2	0.4968	0.7280	5	419
organic feature (ENVO:01000159)	Biofilm	1	0.4968	0.7280	5	419
microbial mat (ENVO:01000008)	Biofilm	3	0.4562	0.7260	3	384

### Validation of post-hoc analysis

Our approach differs from traditional “uninformed” clustering methods, as it identifies EnvO-category enriched clusters in a posthoc analysis. Any (hierarchical) clustering method is suitable in combination. Note that samples from the same environmental category can naturally occur in remote parts of the dendrogram as a result of stochastic assembly processes in some environments or insufficient subcategorization of environmental categories. I.e., this phenomenon is not a short-coming of the clustering process nor the enrichment test. We show that the systematic enrichment test discovers meaningful clusters that would have been otherwise overlooked by classic “uninformed” clustering methods. Compact clusters (low homogeneity/separation ratio) are not necessarily enriched in any EnvO-category (in terms of F-measure). As shown in Supplementary S1 Table, amongst the 245 most compact clusters, only eight have an F-measure above 0.5.

**Fig 7:**
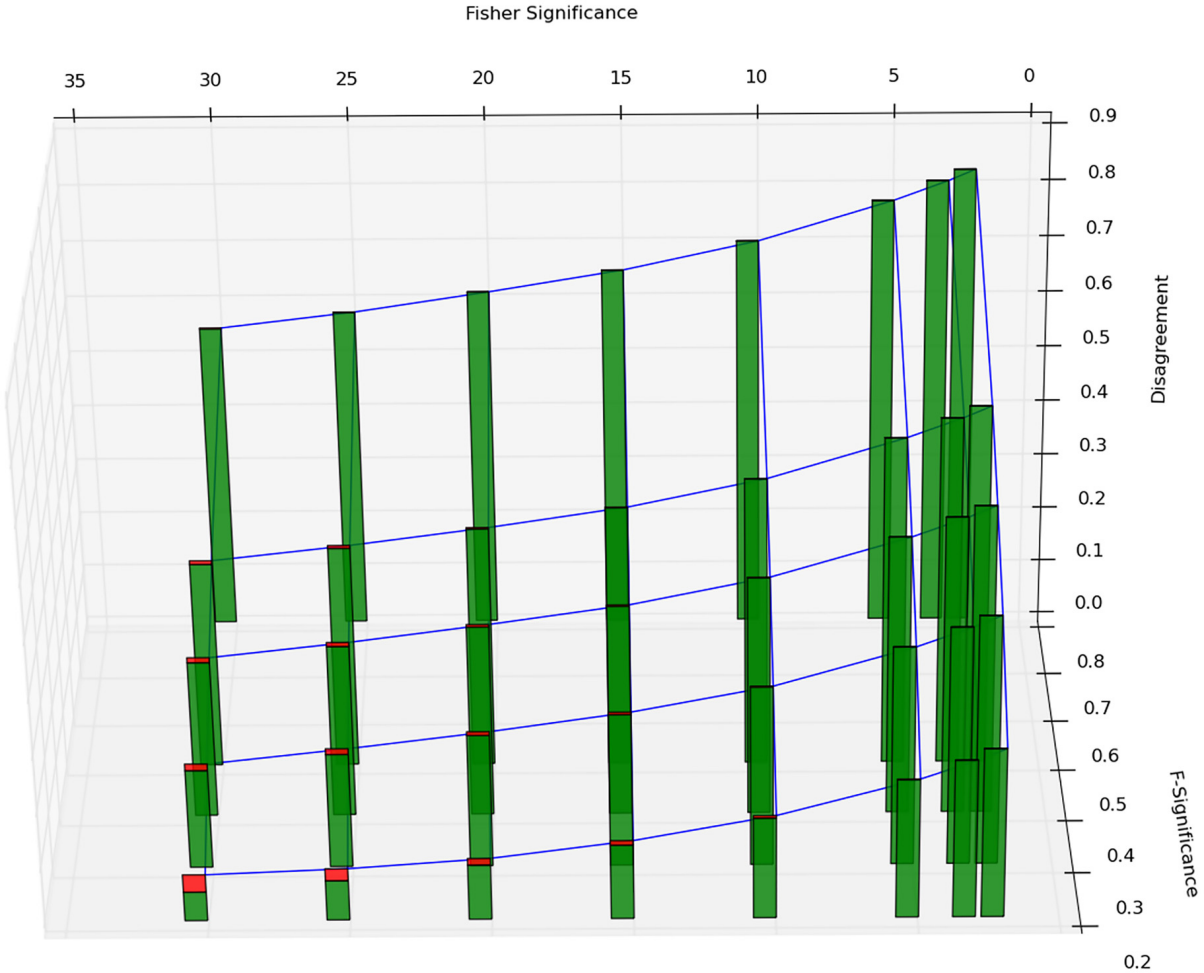
Comparison of Fisher’s exact test and F-measure. We perform a grid search result for various significance thresholds for both tests. The the blue mesh shows disagreement of the tests (in %) and the stacked bars in green and red indicate, respectively, to what extend disagreement stems from Fisher’s exact test claiming signifance but not F-measure and vice versa. Under most commonly used thresholds (−*log*_10_(*p*) score for Fisher’s exact test being 2, 3, or 5) F-measure is a stricter test (completely green bars) as the significant cases are a strict subset of Fisher’s exact test.

We further validate the F-measure in our post-hoc analysis by comparing to the Fisher’s exact test (as used in [19], see Methods). The results are shown in Fig 7. It can be seen that for the commonly used thresholds for Fisher’s exact test (−*log*(*p*) ∈ {2, 3, 5}), the disagreement comes exclusively from cases that are considered significant by Fisher’s exact test but not by F-measure threshold (green bars). In other words, the F-measure is for most thresholds a stricter test, as it only reports a small subset of Fisher’s exact test as significant.

### Salinity as the major driving factor for community assembly?

We revisited the hypothesis postulated in [23], stipulating that salinity largely explains observed patterns of community assembly. In contrast to this, our (much larger) collection of saline samples falls mainly into two clearly separate clusters (see Supplementary S3 Fig), where marine samples and marine sediment samples are the largest contributors, respectively (also cf. Figs 4 and 6b). The observed seafloor/seawater split is in accordance with recent studies on these two realms (e.g. [41]), but our multi-environment contextualization additionally puts these relative differences into a global perspective. Moreover, we observe various outliers. Visual inspection showed that closely related (incl. non-saline) samples in and around the marine sediment cluster are rich in hydrocarbons or molecular nitrogen, thus promoting high relative abundance of Proteobacteria (which are known for their functions in nitrogen fixation and oil-spill related hydrocarbon degradation). We thus hypothesize that these factors are more influential for the assembly of certain communities than salinity.

## Discussion

We here presented a comprehensive effort towards revealing global patterns of beta diversity. To this end we collected a broad range of 16S rRNA profiles of environmental and host associated microbiomes from diverse sources and independent studies. After data integration in a relational database, we showed how to efficiently calculate mutual phylogenetic beta diversity distances (weighted UniFrac) without the information loss in comparison to normal rarefaction. Moreover, our meta-analysis was driven by ontological environmental meta-data information: EnvO-term enriched clusters were automatically detected and used to visualize the emerging global patterns of the Earth meta-community.

Systematically correlating beta diversity dendrograms with environmental annotations is motivated by the idea that oftentimes similar environments select their constituents in similar ways. We also observe samples from the same environment with low homogeneity (i.e. large distances amongst category members). This phenomenon can be either explained by incorrect annotation, by random processes governing assembly or true differences in the same environmental category, which is then instructive for meaningful EnvO subcategorization. On the other hand, it is intriguing to study dense beta diversity clusters with seemingly inconsistent, i.e. diverse environment annotations. Exhaustive scrutiny of environmental parameters might then reveal commonalities amongst those samples and thus explain the low beta diversity. This approach depends on a sufficient, consistent metadata collection for a large set of samples in the future. The systematic integration of metadata into future visualization techniques, as shown here with EnvO-terms, will serve as a new form of hypothesis generation. For example, dense clusters of mixed categories can be explained by latent variables such as high levels of hydrocarbons. If eventually all these potential assembly drivers are consistently captured in the metadata, our method can then be adapted to data mine beyond EnvO annotation. Conversely, exploiting the semantic hierarchy of ontologies, it might also be convenient to extend EnvO to capture over-arching concepts such as “contaminated” or “hydrocarbon-rich” sites. We demonstrated the feasibility of this approach for the analysis of saline vs. non-saline samples.

Occasionally, EnvO mis-annotation or inadequate choice of EnvO-terms incorrectly accounts for mixed clusters. Our method can then point to the source of error and function as an annotation curator, in particular now that our reference dataset is large and robust enough to redline suspecious outliers as demonstrated for the alleged soil microbiome (Fig 6).

With the advent of large comprehensive microbial community databases, we anticipate that it will be possible to provide a context of similarly composed environments for new 16S rRNA profiles, just as BLAST and other sequence comparison tools in conjuction with large sequence databases help to elucidate single sequences. To classify these in a hierarchy (dendrogram) rather than just ranking gives a contextual impression similar to phylogenies for sequences over sequence similarity rankings.

A number of challenges and current limitations prevail, though. Arguably, while the discussed advantages of the chosen components in the proposed pipeline in our opinion justify their application, we also note their caveats here and mention potential alternatives. Cross-plattform meta analyses have to deal with inherent protocol and technology biases: DNA extraction kits and sequencing platforms are afflicted with specific errors, different variable regions differ in informative power to distinguish phylogenetic clades, so the choice of primers impacts relative OTU abundances. Additionally, alignment quality for reference phylogenies and differences in sequence filtering can skew beta diversity calculations [42]. Environment Ontology annotations are far from being perfect, as neither automatic nor manual methods guarantee 100% accuracy. OTU clustering methods often produce surprisingly different results, as argued in [43]. Closed-reference OTU picking discards all sequences that do not match a provided reference (GreenGenes in our case) and thus represents the original sample inaccurately, especially in understudied environments with high diversity such as soils. Alternatively, open-reference or de novo OTU clustering could be employed, yet this is a daunting task with its own caveats: the considerably higher computational effort is less parallelizable, the number of comparable samples is restricted by the choice of 16S rRNA region and the lack of a high-quality phylogeny based on full sequences severely impedes downstream phylogenetic beta diversity calculation. The latter can be addressed by resorting to non-phylogenetic beta diversity but that would disregard evolutionary relatedness of OTUs. Moreover, as pointed out in [7], short reads stretching only over one or few variable regions might be unsuitable for de novo OTU picking.

Rarefaction introduces a loss of information, and albeit substantially reduced, it still persists with our proposed method of adaptive rarefaction. One concern regarding the generality of our results is due to the sampling bias induced by very large scale studies appearing as sole contributors to a certain environment. As can be seen in the interactive coloring of PCoA plots (ecophyl.info/html/SI), the ecosystem distribution and particularly EnvO-distribution is similar to study distribution. Beta diversity patterns could thus be explained to some extend by study methods (and possibly systematic artefacts). However, many independent studies do confirm characteristic beta diversity distributions, especially for soils and human-associated samples, when including small scale studies. Our visualization techniques reflect this important measure of confidence.

Likewise, some large studies like high resolution time series can give a false impression of community similarity (i.e, tight clustering in PCoA and UPGMA plots) in a certain environment, if a single study dominates the contribution. As a remedy, we would be tempted to downsample on study level, i.e. select only few representative samples for each study. However, genuine beta diversity has been captured in large scale studies such as [44], so that simple exclusion of samples from large studies will lead to loss of important information. In addition to uneven sample size, the aspect of uneven study size should be addressed by future meta analyses. The environmental equivalents of enterotypes (clusters of gut microbiomes and conceptually extended in [17] to all human-associated environments), are barely observable throughout the entirety of samples used in our meta-analysis. In accordance with Koren et al [17], we detect gradients of microbiomes for nearly all ecosystems. Compact clusters with high homogeneity/separation ratio often only exist when single studies constitute the bulk of an ecosystem (or -subsystem) and thus require further evidence to be considered an “environmental enterotype”.

Our generated beta diversity maps also suggest that many studies still tap into unchartered territory, indicating that the beta diversity space for the entirety of Earth microbiomes is far from being fully explored. Despite our best attempts to collect a comprehensive dataset, various samples still appear rather unique. Note that the large collection of millions of pairwise beta diversities was a prerequisite to develop a sense of uniqueness. However, increased coverage of similar environments in the future will inevitably either improve confidence in uniqueness or find closer matches. The effect of microbiome uniqueness is exacerbated, when we start including OTUs beyond closed references.

Systematic errors specific to studies and/or sequencing technologies are still a concern. We therefore report in our final result table the number of studies that support a cluster enrichment and developed an interactive visualization that allows to inspect PCoA clusters color-coded by EnvO-annotation, by ecosystem or by study. Despite the discussed limitations of cross-study meta analyses, we believe that by integrating as many samples as possible, a global picture of diversity is emerging due the law of large numbers. We are encouraged by a number of similar (albeit substantially smaller) works corroborating the observation that samples do cluster by environment rather than just by study [7, 21, 22].

## Supporting Information

S1 Fig Alpha diversity box plots for different EnvO soil types. Farm soil, grassland and marine sediments appear as most diverse soil types. Note that “Forest” appears low due to misannotations of many low-diversity insect-associated communities, see also Fig 6.
(TIFF)

S2 Fig SQL Database diagram of all tables. (TIFF)

S3 Fig Dendrogram of saline (black in dendrogram) and non-saline samples (red in dendrogram) using adaptive rarefaction/UniFrac/UPGMA. Saline samples do not form a single cohesive cluster, as previous findings suggested but are rather split into two main clusters and several outliers. Note that one cluster (containing polluted marine sediments and marine oil spill samples) contains non-saline samples with high levels of hydrocarbons, suggesting that this is the major driving force for this cluster.
(TIFF)

S4 Fig Repeated beta diversity results for static and adaptive rarefaction in combination with Weighted UniFrac and Principle Coordinate Analysis. Large uncertainty ellipsoids in the static rarefaction based plot in **(b)** show relative positions of samples in the three-dimensional space spanned by the first three Principal Components are less confined than in the case of adaptive rarefaction **(a)**. Note that Principle coordinates were calculated independently, which leads to different orientations in **(a)** and **(b)**. The averaged variances of beta diversity distance matrices also clearly show lower values for the case of Adaptive Rarefaction as compared to conventional rarefaction **(c)**.
(TIFF)

S5 Fig Evaluation of the EnvO annotation algorithm. We define the distance between a predicted and a manually added EnvO-term as the shortest path in the undirected EnvO Graph. As can be seen, most automated annotations are exact matches (distance 0) or not more than one step away in the EnvO graph (distance 1).
(TIFF)

S1 Table Clusters selected based on homogeneity/separation ratio. The table, which is sorted by homogeneity/separation ratio, contains only few significantly enriched clusters (those with *F* > 0.5 are shown in bold).
(PDF)

S2 Table Number of dropped sequences from inhouse samples. (PDF)
