# Supplementary figures and images for: Comprehensive Meta-analysis of Ontology Annotated 16S rRNA Profiles Identifies Beta Diversity Clusters of Environmental Bacterial Communities
Source: PLoS Comput Biol. 2015 Oct 12;11(10):e1004468. doi: 10.1371/journal.pcbi.1004468 (PMC4601763; doi:10.1371/journal.pcbi.1004468)

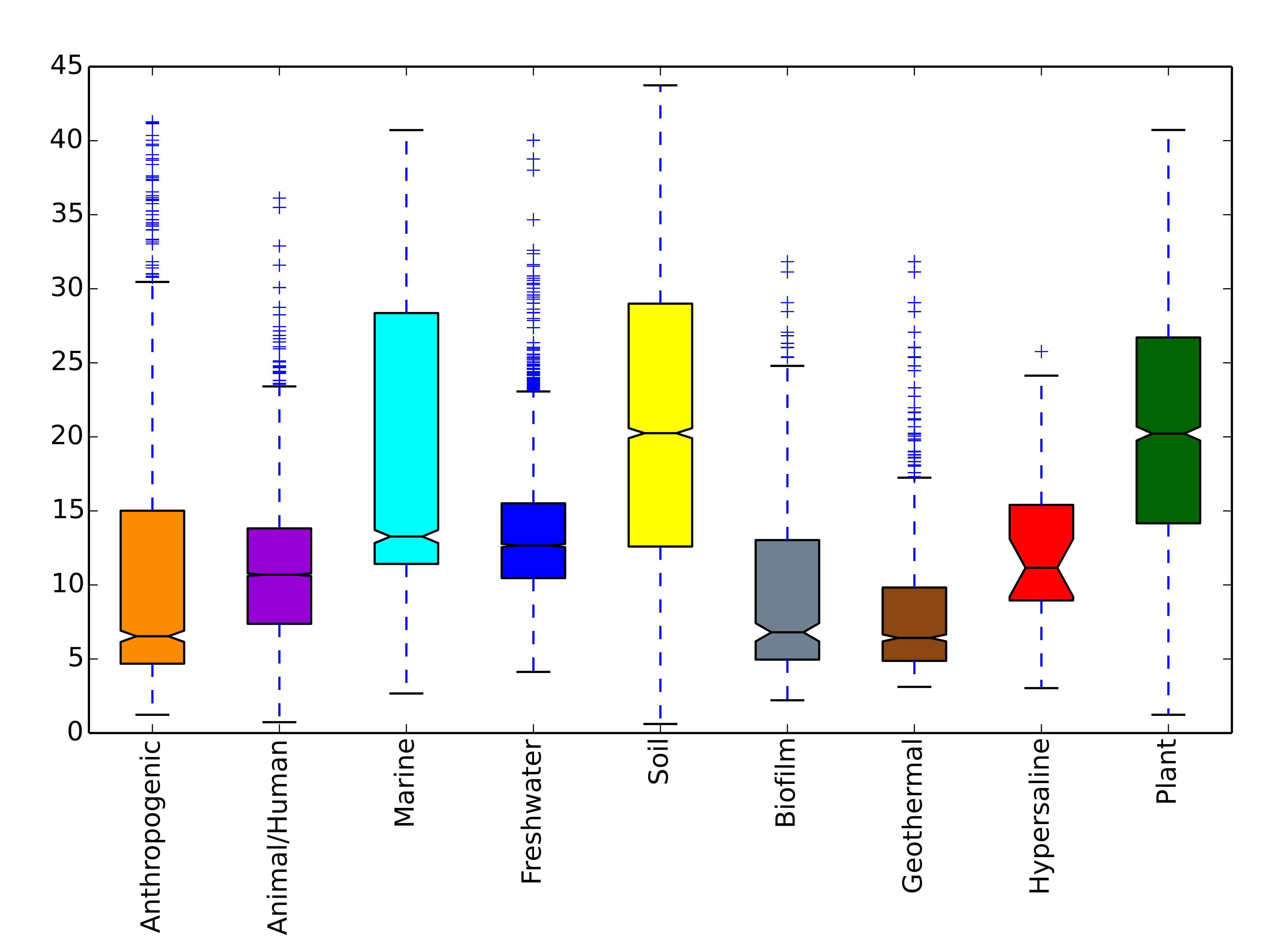

Supplement: S1 Fig — Farm soil, grassland and marine sediments appear as most diverse soil types. Note that “Forest” appears low due to misannotations of many low-diversity insect-associated communities, see also Fig 6. (TIFF) [file pcbi.1004468.s001.tiff]

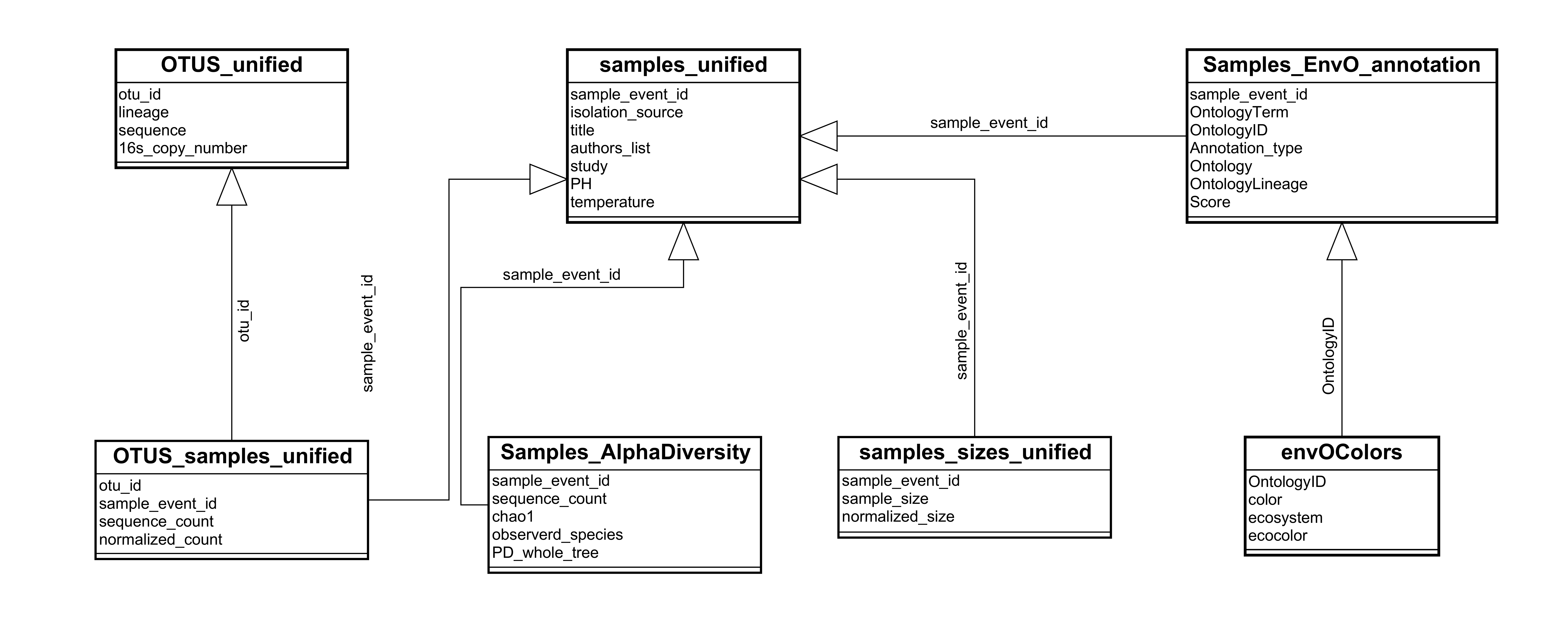

Supplement: S2 Fig — (TIFF) [file pcbi.1004468.s002.tiff]

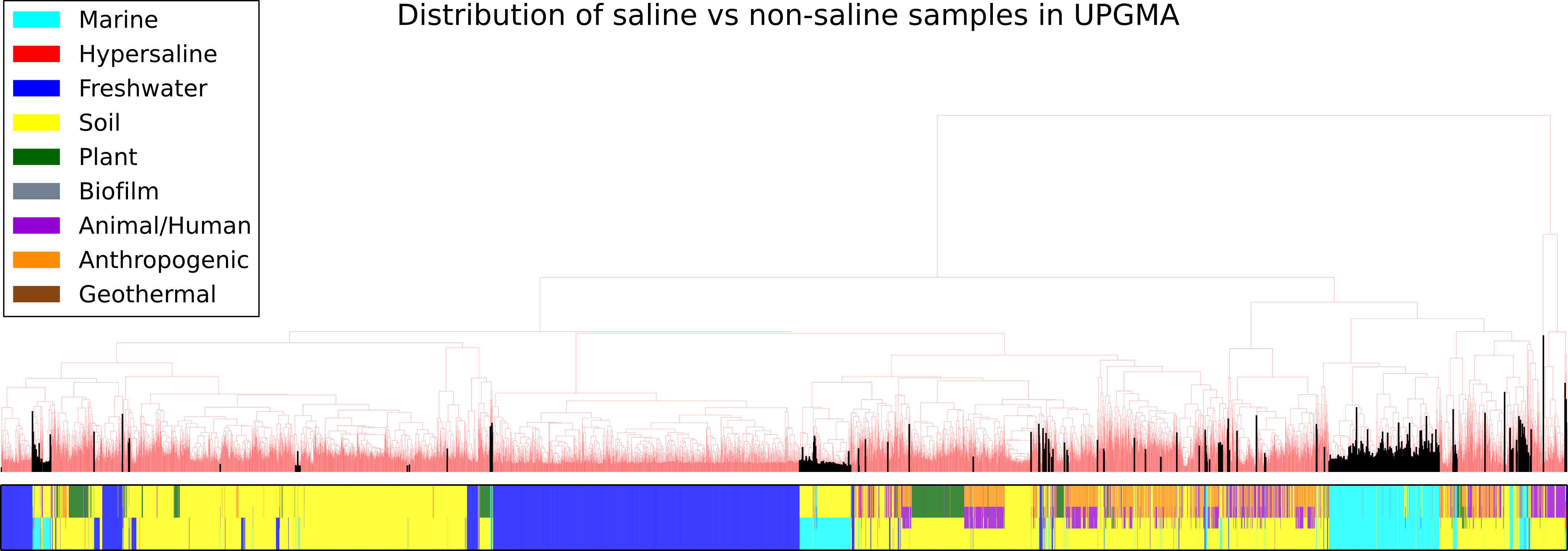

Supplement: S3 Fig — Saline samples do not form a single cohesive cluster, as previous findings suggested but are rather split into two main clusters and several outliers. Note that one cluster (containing polluted marine sediments and marine oil spill samples) contains non-saline samples with high levels of hydrocarbons, suggesting that this is the major driving force for this cluster. (TIFF) [file pcbi.1004468.s003.tiff]

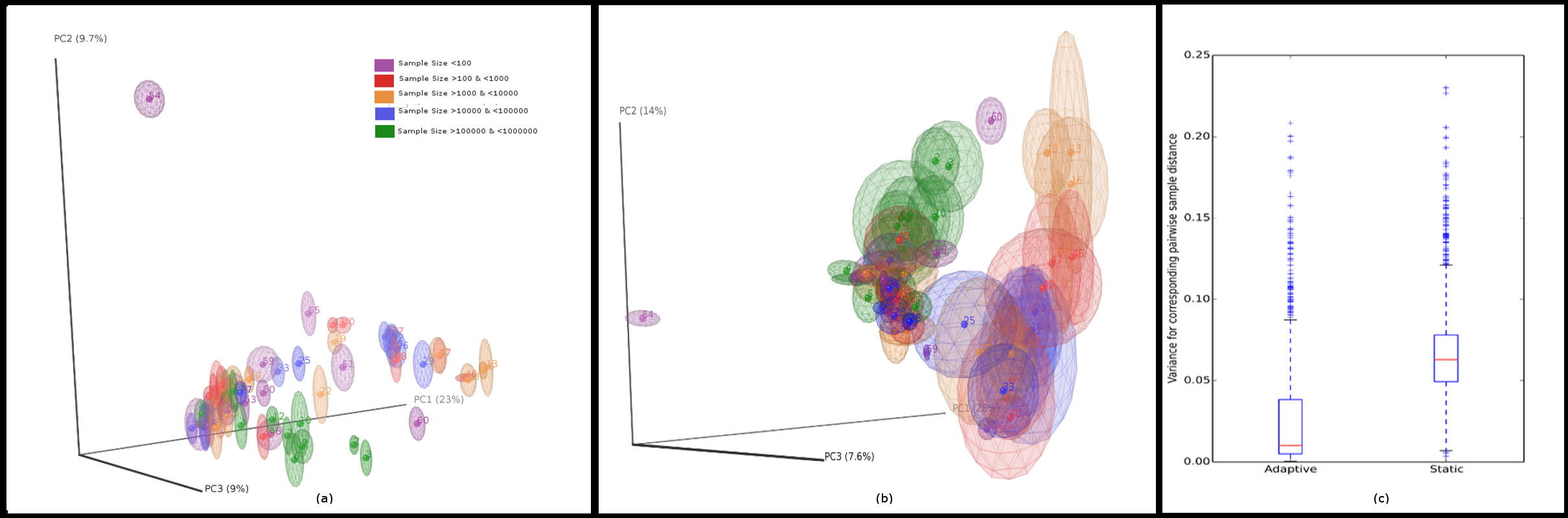

Supplement: S4 Fig — Large uncertainty ellipsoids in the static rarefaction based plot in (b) show relative positions of samples in the three-dimensional space spanned by the first three Principal Components are less confined than in the case of adaptive rarefaction (a). Note that Principle coordinates were calculated independently, which leads to different orientations in (a) and (b). The averaged variances of beta diversity distance matrices also clearly show lower values for the case of Adaptive Rarefaction as compared to conventional rarefaction (c). (TIFF) [file pcbi.1004468.s004.tiff]

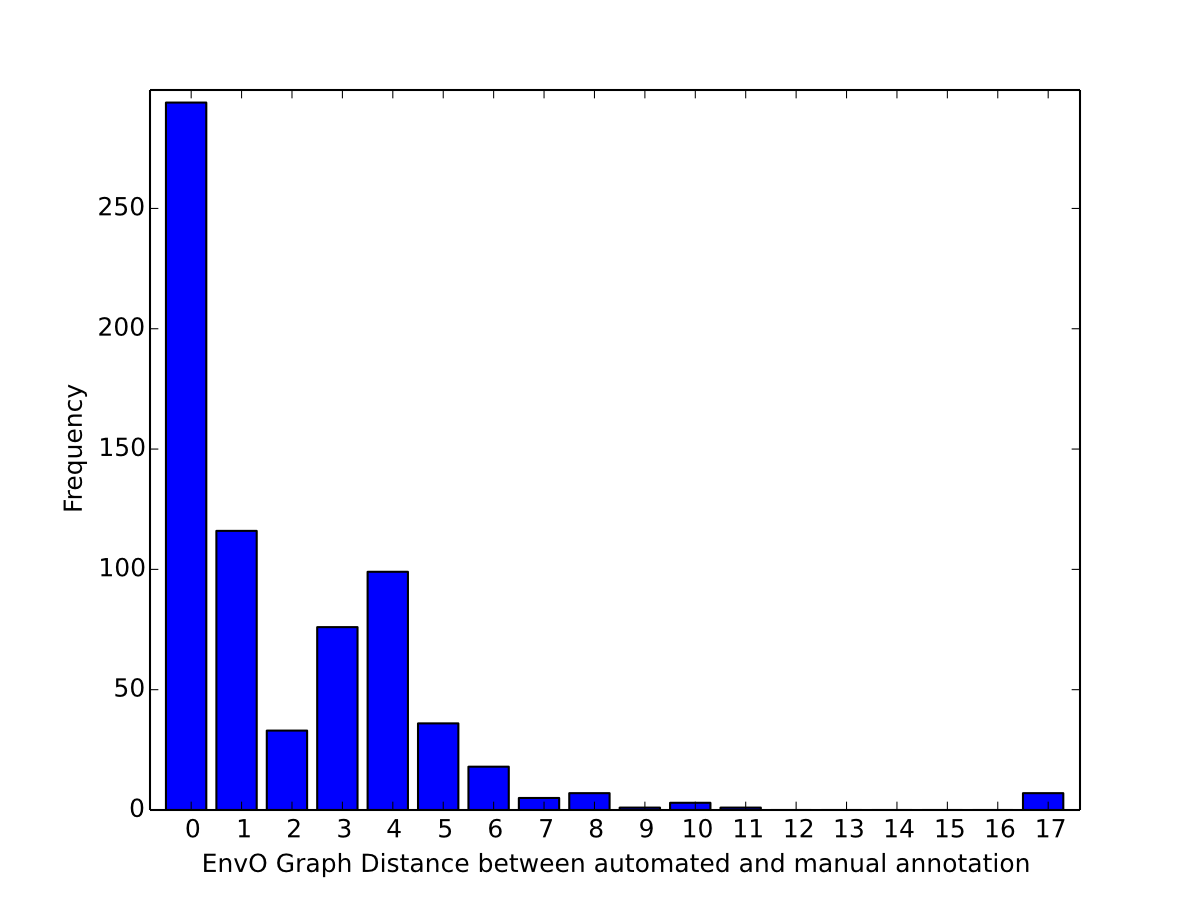

Supplement: S5 Fig — We define the distance between a predicted and a manually added EnvO-term as the shortest path in the undirected EnvO Graph. As can be seen, most automated annotations are exact matches (distance 0) or not more than one step away in the EnvO graph (distance 1). (TIFF) [file pcbi.1004468.s005.tiff]
