# Supplementary material for: Comprehensive Meta-analysis of Ontology Annotated 16S rRNA Profiles Identifies Beta Diversity Clusters of Environmental Bacterial Communities
Source: PLoS Comput Biol. 2015 Oct 12;11(10):e1004468. doi: 10.1371/journal.pcbi.1004468 (PMC4601763; doi:10.1371/journal.pcbi.1004468)
Supplement: S1 Table — The table, which is sorted by homogeneity/separation ratio, contains only few significantly enriched clusters (those with F > 0.5 are shown in bold). (PDF) [file pcbi.1004468.s006.pdf]

| EnvoID | Cluster size | Envo Total | Supported Studies | DAG-Level Envo-Term | Precision | Recall | F | Homogeneity | Separation | Hom/Sep |
|---|---|---|---|---|---|---|---|---|---|---|
| ***ENVO:02000040*** | ***135*** | ***346*** | ***4*** | ***03 mucus*** | ***0.985185*** | ***0.384393*** | ***0.553015*** | ***0.074931*** | ***0.799402*** | ***0.09373382*** |
| ***ENVO:00005791*** | ***12*** | ***23*** | ***1*** | ***02 sterile water*** | ***0.833333*** | ***0.434783*** | ***0.571429*** | ***0.099618*** | ***0.727716*** | ***0.13689131*** |
| ENVO:00009001 | 185 | 719 | 9 | 03 plant-associated habitat | 0.789189 | 0.20306 | 0.323009 | 0.125229 | 0.814646 | 0.15372198 |
| ***ENVO:00005791*** | ***13*** | ***23*** | ***1*** | ***02 sterile water*** | ***0.769231*** | ***0.434783*** | ***0.555556*** | ***0.114648*** | ***0.725085*** | ***0.15811663*** |
| ENVO:00005778 | 39 | 26 | 3 | 02 tropical soil | 0.666667 | 1 | 0.8 | 0.113092 | 0.711382 | 0.15897507 |
| ENVO:00000111 | 16 | 81 | 1 | 04 forest | 1 | 0.197531 | 0.329897 | 0.134909 | 0.822616 | 0.16399997 |
| ***ENVO:00005791*** | ***14*** | ***23*** | ***2*** | ***02 sterile water*** | ***0.714286*** | ***0.434783*** | ***0.540541*** | ***0.12811*** | ***0.722983*** | ***0.17719642*** |
| ENVO:00002003 | 32 | 2462 | 2 | 04 feces | 1 | 0.012998 | 0.025662 | 0.134548 | 0.743301 | 0.18101415 |
| ENVO:00000077 | 24 | 108 | 11 | 03 agricultural feature | 0.166667 | 0.037037 | 0.060606 | 0.136926 | 0.743367 | 0.18419704 |
| ENVO:00002003 | 17 | 2462 | 4 | 04 feces | 1 | 0.006905 | 0.013715 | 0.147539 | 0.799507 | 0.18453747 |
| ENVO:00000879 | 87 | 75 | 12 | 03 Tropical and subtropical moist broadleaf forest bi | 0.172414 | 0.2 | 0.185185 | 0.148286 | 0.796282 | 0.18622297 |
| ***ENVO:00000086*** | ***14*** | ***5*** | ***2*** | ***03 plain*** | ***0.357143*** | ***1*** | ***0.526316*** | ***0.158067*** | ***0.843048*** | ***0.18749466*** |
| ENVO:00002003 | 11 | 2462 | 5 | 04 feces | 1 | 0.004468 | 0.008896 | 0.132742 | 0.697767 | 0.19023829 |
| ENVO:00000247 | 13 | 361 | 1 | 04 volcano | 1 | 0.036011 | 0.069519 | 0.148884 | 0.781639 | 0.19047668 |
| ENVO:00000247 | 15 | 361 | 1 | 04 volcano | 1 | 0.041551 | 0.079787 | 0.144803 | 0.7576 | 0.19113384 |
| ENVO:00000172 | 15 | 2 | 2 | 04 sandy desert | 0.066667 | 0.5 | 0.117647 | 0.126731 | 0.659634 | 0.19212321 |
| ENVO:00000878 | 19 | 460 | 6 | 03 Mediterranean forests, woodlands, and shrub biome | 0.526316 | 0.021739 | 0.041754 | 0.142494 | 0.735725 | 0.19367834 |
| ENVO:00000077 | 26 | 108 | 11 | 03 agricultural feature | 0.153846 | 0.037037 | 0.059701 | 0.147141 | 0.741016 | 0.19856656 |
| ENVO:00000878 | 21 | 460 | 1 | 03 Mediterranean forests, woodlands, and shrub biome | 1 | 0.045652 | 0.087318 | 0.145776 | 0.733967 | 0.19861383 |
| ENVO:02000036 | 11 | 430 | 5 | 03 saliva | 0.363636 | 0.009302 | 0.018141 | 0.141805 | 0.711569 | 0.19928496 |
| ENVO:00000447 | 19 | 368 | 2 | 02 marine biome | 0.842105 | 0.043478 | 0.082687 | 0.15822 | 0.792486 | 0.19965021 |
| ENVO:00000879 | 96 | 75 | 14 | 03 Tropical and subtropical moist broadleaf forest bi | 0.15625 | 0.2 | 0.175439 | 0.160004 | 0.792882 | 0.20180052 |
| ENVO:02000037 | 34 | 558 | 4 | 03 sebum | 0.794118 | 0.048387 | 0.091216 | 0.147276 | 0.723308 | 0.2036145 |
| ENVO:00002003 | 23 | 2462 | 7 | 04 feces | 0.782609 | 0.007311 | 0.014487 | 0.175598 | 0.861156 | 0.20390963 |
| ENVO:02000040 | 24 | 346 | 1 | 03 mucus | 0.958333 | 0.066474 | 0.124324 | 0.150412 | 0.734158 | 0.20487688 |
| ENVO:0010013 | 31 | 14 | 6 | 06 fermented cereal beverage | 0.16129 | 0.357143 | 0.222222 | 0.153422 | 0.748733 | 0.20490883 |
| ENVO:00000878 | 22 | 460 | 1 | 03 Mediterranean forests, woodlands, and shrub biome | 1 | 0.047826 | 0.091286 | 0.15091 | 0.732163 | 0.2061153 |
| ENVO:00000039 | 31 | 258 | 1 | 05 fjord | 1 | 0.120155 | 0.214533 | 0.147837 | 0.715912 | 0.20650164 |
| ENVO:00000447 | 16 | 368 | 2 | 02 marine biome | 0.9375 | 0.040761 | 0.078125 | 0.152935 | 0.740292 | 0.2065874 |
| ENVO:00002003 | 14 | 2462 | 4 | 04 feces | 0.928571 | 0.00528 | 0.010501 | 0.170472 | 0.82175 | 0.20744995 |
| ENVO:00000247 | 15 | 361 | 1 | 04 volcano | 1 | 0.041551 | 0.079787 | 0.16115 | 0.775678 | 0.20775373 |
| ENVO:00002003 | 12 | 2462 | 3 | 04 feces | 1 | 0.004874 | 0.009701 | 0.146113 | 0.702623 | 0.20795363 |
| ENVO:00000039 | 16 | 258 | 1 | 05 fjord | 1 | 0.062016 | 0.116788 | 0.155799 | 0.747362 | 0.20846524 |
| ENVO:00000086 | 17 | 5 | 3 | 03 plain | 0.294118 | 1 | 0.454545 | 0.175088 | 0.839395 | 0.20858833 |
| ENVO:00002003 | 72 | 2462 | 2 | 04 feces | 1 | 0.029245 | 0.056827 | 0.163675 | 0.780197 | 0.20978676 |
| ENVO:02000040 | 13 | 346 | 2 | 03 mucus | 0.538462 | 0.020231 | 0.038997 | 0.163788 | 0.780726 | 0.20978935 |
| ENVO:02000037 | 11 | 558 | 2 | 03 sebum | 0.636364 | 0.012545 | 0.024605 | 0.138449 | 0.65809 | 0.21038004 |
| ENVO:00002003 | 13 | 2462 | 3 | 04 feces | 1 | 0.00528 | 0.010505 | 0.177087 | 0.836993 | 0.21157525 |
| ENVO:00000878 | 23 | 460 | 2 | 03 Mediterranean forests, woodlands, and shrub biome | 0.956522 | 0.047826 | 0.091097 | 0.155955 | 0.732278 | 0.2129724 |
| ENVO:00000426 | 15 | 206 | 2 | 03 ocean floor | 1 | 0.072816 | 0.135747 | 0.141413 | 0.663153 | 0.2132434 |
| ENVO:00000878 | 42 | 460 | 2 | 03 Mediterranean forests, woodlands, and shrub biome | 0.952381 | 0.086957 | 0.159363 | 0.148404 | 0.694508 | 0.2136822 |
| ENVO:00005801 | 24 | 444 | 1 | 02 rhizosphere | 1 | 0.054054 | 0.102564 | 0.155046 | 0.725062 | 0.21383826 |
| ENVO:00000173 | 45 | 100 | 1 | 04 rocky desert | 0.977778 | 0.44 | 0.606897 | 0.166041 | 0.772718 | 0.21487917 |
| ENVO:00002003 | 11 | 2462 | 2 | 04 feces | 1 | 0.004468 | 0.008896 | 0.156508 | 0.727393 | 0.21516292 |
| ENVO:00000447 | 11 | 368 | 1 | 02 marine biome | 1 | 0.029891 | 0.058047 | 0.15977 | 0.741828 | 0.21537337 |
| ENVO:00002003 | 24 | 2462 | 4 | 04 feces | 0.958333 | 0.009342 | 0.018504 | 0.166257 | 0.770564 | 0.21576014 |
| ENVO:00002003 | 11 | 2462 | 1 | 04 feces | 1 | 0.004468 | 0.008896 | 0.156793 | 0.723658 | 0.21666727 |
| ENVO:00002003 | 11 | 2462 | 2 | 04 feces | 1 | 0.004468 | 0.008896 | 0.154225 | 0.708213 | 0.21776641 |
| ENVO:00002003 | 12 | 2462 | 4 | 04 feces | 1 | 0.004874 | 0.009701 | 0.165016 | 0.754948 | 0.21857929 |
| ENVO:00009001 | 50 | 719 | 2 | 03 plant-associated habitat | 0.94 | 0.065369 | 0.122237 | 0.164001 | 0.747901 | 0.2192817 |
| ENVO:00000247 | 70 | 361 | 1 | 04 volcano | 1 | 0.193906 | 0.324826 | 0.17315 | 0.786891 | 0.22004318 |
| ENVO:00000446 | 19 | 2528 | 1 | 01 terrestrial biome | 1 | 0.007516 | 0.01492 | 0.164695 | 0.746838 | 0.22052306 |
| ENVO:00002003 | 24 | 2462 | 7 | 04 feces | 0.958333 | 0.009342 | 0.018504 | 0.163778 | 0.7423 | 0.22063586 |
| ENVO:00002003 | 13 | 2462 | 1 | 04 feces | 1 | 0.00528 | 0.010505 | 0.160871 | 0.728579 | 0.22080104 |
| ENVO:00002003 | 20 | 2462 | 4 | 04 feces | 0.85 | 0.006905 | 0.013699 | 0.161392 | 0.724493 | 0.22276544 |
| ENVO:02000037 | 54 | 558 | 5 | 03 sebum | 0.87037 | 0.084229 | 0.153595 | 0.166985 | 0.745493 | 0.22399271 |
| ENVO:00002003 | 30 | 2462 | 4 | 04 feces | 0.966667 | 0.011779 | 0.023274 | 0.150009 | 0.668969 | 0.22423909 |
| ENVO:00000358 | 16 | 2 | 6 | 04 protected area | 0.125 | 1 | 0.222222 | 0.158808 | 0.707841 | 0.22435547 |
| ENVO:00000572 | 11 | 171 | 1 | 02 subterrestrial habitat | 1 | 0.064327 | 0.120879 | 0.150091 | 0.66337 | 0.22625533 |
| ENVO:00000447 | 12 | 368 | 1 | 02 marine biome | 1 | 0.032609 | 0.063158 | 0.167542 | 0.739786 | 0.2264736 |
| ENVO:02000037 | 172 | 558 | 6 | 03 sebum | 0.55814 | 0.172043 | 0.263014 | 0.157324 | 0.691694 | 0.2274474 |
| ENVO:0010013 | 36 | 14 | 6 | 06 fermented cereal beverage | 0.138889 | 0.357143 | 0.2 | 0.170273 | 0.746904 | 0.22797173 |
| ENVO:00002003 | 12 | 2462 | 1 | 04 feces | 1 | 0.004874 | 0.009701 | 0.165588 | 0.72514 | 0.22835315 |
| ENVO:02000037 | 56 | 558 | 5 | 03 sebum | 0.857143 | 0.086022 | 0.156352 | 0.17109 | 0.744443 | 0.22982283 |
| ENVO:00000039 | 20 | 258 | 1 | 05 fjord | 1 | 0.077519 | 0.143885 | 0.172148 | 0.747512 | 0.23029463 |
| ENVO:00000173 | 13 | 100 | 1 | 04 rocky desert | 0.923077 | 0.12 | 0.212389 | 0.169421 | 0.734802 | 0.23056687 |
| ENVO:02000037 | 175 | 558 | 7 | 03 sebum | 0.56 | 0.175627 | 0.267394 | 0.159542 | 0.69049 | 0.23105621 |
| ENVO:00002003 | 37 | 2462 | 1 | 04 feces | 1 | 0.015028 | 0.029612 | 0.138211 | 0.596896 | 0.23154955 |
| ENVO:00000021 | 70 | 1614 | 1 | 03 freshwater lake | 1 | 0.043371 | 0.083135 | 0.157725 | 0.680419 | 0.2318057 |
| ENVO:02000037 | 176 | 558 | 7 | 03 sebum | 0.556818 | 0.175627 | 0.26703 | 0.160315 | 0.689915 | 0.2323692 |
| ENVO:02000037 | 56 | 558 | 1 | 03 sebum | 0.589286 | 0.05914 | 0.107492 | 0.155964 | 0.670829 | 0.23249442 |
| ENVO:00002003 | 12 | 2462 | 3 | 04 feces | 1 | 0.004874 | 0.009701 | 0.164965 | 0.703065 | 0.23463691 |
| ENVO:00000447 | 21 | 368 | 1 | 02 marine biome | 1 | 0.057065 | 0.107969 | 0.180049 | 0.766827 | 0.23479742 |
| ENVO:00000039 | 107 | 258 | 1 | 05 fjord | 1 | 0.414729 | 0.586301 | 0.169063 | 0.719435 | 0.23499413 |
| ENVO:00000062 | 21 | 102 | 4 | 03 populated place | 0.571429 | 0.117647 | 0.195122 | 0.162281 | 0.689921 | 0.23521679 |
| ENVO:02000036 | 17 | 430 | 3 | 03 saliva | 0.529412 | 0.02093 | 0.040268 | 0.185219 | 0.781772 | 0.23692202 |
| ENVO:02000040 | 12 | 346 | 3 | 03 mucus | 0.833333 | 0.028902 | 0.055866 | 0.166449 | 0.701278 | 0.23735095 |
| ID:0000002 | 90 | 2473 | 4 | 04 gut | 1 | 0.036393 | 0.07023 | 0.171506 | 0.720927 | 0.23789649 |
| ENVO:00002003 | 23 | 2462 | 5 | 04 feces | 1 | 0.009342 | 0.018511 | 0.164908 | 0.691062 | 0.23862982 |
| ENVO:02000040 | 11 | 346 | 3 | 03 mucus | 0.454545 | 0.014451 | 0.028011 | 0.165392 | 0.691912 | 0.23903618 |
| ENVO:00000021 | 67 | 1614 | 1 | 03 freshwater lake | 1 | 0.041512 | 0.079714 | 0.163927 | 0.684807 | 0.23937693 |
| ENVO:00002003 | 46 | 2462 | 8 | 04 feces | 0.934783 | 0.017465 | 0.03429 | 0.192611 | 0.803935 | 0.23958529 |
| ENVO:00002116 | 20 | 28 | 4 | 02 contaminated soil | 0.15 | 0.107143 | 0.125 | 0.172045 | 0.71584 | 0.24034002 |
| ENVO:02000036 | 11 | 430 | 6 | 03 saliva | 0.818182 | 0.02093 | 0.040816 | 0.166972 | 0.69325 | 0.24085395 |
| ENVO:00000878 | 28 | 460 | 2 | 03 Mediterranean forests, woodlands, and shrub biome | 0.964286 | 0.058696 | 0.110656 | 0.175398 | 0.728073 | 0.24090716 |
| ENVO:00000247 | 54 | 361 | 1 | 04 volcano | 1 | 0.149584 | 0.260241 | 0.181437 | 0.75261 | 0.24107705 |
| ENVO:00002003 | 11 | 2462 | 3 | 04 feces | 1 | 0.004468 | 0.008896 | 0.157794 | 0.654457 | 0.24110675 |
| ENVO:00000134 | 25 | 66 | 2 | 04 permafrost | 0.96 | 0.363636 | 0.527473 | 0.152372 | 0.629634 | 0.24200091 |
| ENVO:00002150 | 16 | 145 | 1 | 04 coastal water | 1 | 0.110345 | 0.198758 | 0.177443 | 0.732549 | 0.2422268 |
| ENVO:00002992 | 41 | 32 | 5 | 03 Temperate needle-leaf forests or woodlands | 0.414634 | 0.53125 | 0.465753 | 0.169558 | 0.69939 | 0.24243698 |
| ENVO:00000247 | 94 | 361 | 1 | 04 volcano | 1 | 0.260388 | 0.413187 | 0.190093 | 0.783215 | 0.24270858 |
| ENVO:00000021 | 108 | 1614 | 1 | 03 freshwater lake | 1 | 0.066914 | 0.125436 | 0.164996 | 0.679606 | 0.24278185 |
| ENVO:00000874 | 13 | 109 | 2 | 03 Tundra biome | 0.846154 | 0.100917 | 0.180328 | 0.163429 | 0.670582 | 0.24371218 |
| ENVO:00000426 | 19 | 206 | 2 | 03 ocean floor | 1 | 0.092233 | 0.168889 | 0.160717 | 0.658897 | 0.24391825 |
| ENVO:02000040 | 18 | 346 | 3 | 03 mucus | 0.5 | 0.026012 | 0.049451 | 0.179078 | 0.732457 | 0.24448944 |
| ENVO:00000173 | 23 | 100 | 1 | 04 rocky desert | 1 | 0.23 | 0.373984 | 0.178917 | 0.730954 | 0.2447719 |
| ENVO:00000039 | 72 | 258 | 1 | 05 fjord | 1 | 0.27907 | 0.436364 | 0.18315 | 0.747638 | 0.2449715 |
| ENVO:00005806 | 11 | 7 | 6 | 03 Tropical and subtropical coniferous forest biome | 0.090909 | 0.142857 | 0.111111 | 0.187188 | 0.764007 | 0.24500823 |
| ENVO:00000021 | 17 | 1614 | 1 | 03 freshwater lake | 1 | 0.010533 | 0.020846 | 0.164714 | 0.669231 | 0.24612428 |
| ENVO:02000037 | 11 | 558 | 3 | 03 sebum | 0.909091 | 0.017921 | 0.035149 | 0.166775 | 0.676033 | 0.24669654 |
| ENVO:00000021 | 52 | 1614 | 1 | 03 freshwater lake | 1 | 0.032218 | 0.062425 | 0.17381 | 0.703844 | 0.24694393 |
| ENVO:00009001 | 63 | 719 | 2 | 03 plant-associated habitat | 0.952381 | 0.083449 | 0.153453 | 0.182306 | 0.737762 | 0.24710679 |
| ENVO:00000062 | 23 | 102 | 4 | 03 populated place | 0.521739 | 0.117647 | 0.192 | 0.170298 | 0.688795 | 0.24724047 |
| ENVO:00000021 | 11 | 1614 | 1 | 03 freshwater lake | 1 | 0.006815 | 0.013538 | 0.167594 | 0.677521 | 0.24736355 |

**Table 1. Clusters selected based on homogeneity/separation ratio.** The table, which is sorted by homogeneity/separation ratio, contains only few significantly enriched clusters (those with $F > 0.5$ are shown in bold).
