# Supplementary material for: Comprehensive Meta-analysis of Ontology Annotated 16S rRNA Profiles Identifies Beta Diversity Clusters of Environmental Bacterial Communities
Source: PLoS Comput Biol. 2015 Oct 12;11(10):e1004468. doi: 10.1371/journal.pcbi.1004468 (PMC4601763; doi:10.1371/journal.pcbi.1004468)
Supplement: S2 Table — (PDF) [file pcbi.1004468.s007.pdf]

Study	Samples	Total Sequence Count	Total Sequences Clustered against Green genes	Not Clustered against Green genes
Mangroves	5	321317	211353	109964
Waste water	6	365782	243704	122078
Soil	10	158184	64540	93644
Salt flat	3	205174	172295	32879
Crab gut	6	947847	462878	484969
**Total**	30	1998304	1154770	843534
**Percentage %**			57.7875038	42.2124962

Table 1. Number of dropped sequences from inhouse samples.
